# Engineering a newly identified alcohol dehydrogenase from *Sphingobium* Sp. for efficient utilization of nicotinamide cofactors biomimetics

**DOI:** 10.1186/s40643-025-00870-z

**Published:** 2025-05-05

**Authors:** Yichun Zhu, Jieyu Zhou, Xiangyuan Gu, Huiru Wang, Hao Han, Ye Ni

**Affiliations:** Key laboratory of industrial Biotechnology, School of Biotechnology, Ministry of Education, Jiangnan University, Wuxi, 214122 Jiangsu China

**Keywords:** Alcohol dehydrogenase, Nicotinamide cofactor biomimetics (NCBs), Syringyl alcohol, Semi-rational engineering, Cofactor specificity

## Abstract

Nicotinamide cofactor biomimetics (NCBs) serve as low-cost alternatives to the expensive NAD(P)^+^/NAD(P)H, holding significant potential for applications in oxidoreductases. In this study, an alcohol dehydrogenase (*Sp*ADH2) from *Sphingobium* sp. SYK-6 was identified for the utilization of synthetic NCBs. *Sp*ADH2 exhibited a catalytic activity of 11.55 U/g in oxidation of syringyl alcohol when utilizing *para*-3-carbamoyl-1-(4-carboxybenzyl)pyridin-1-ium (*p*-BANA^+^) as cofactor. Semi-rational engineering of *Sp*ADH2 led to identification of key variants (H43L, A290I, H43L/A290I) with enhanced catalytic efficiency and specificity using *p*-BANA^+^ as the cofactor. Compared with wild-type, variant H43L/A290I exhibited a 7-fold increase in activity and an astonishing 6750-fold improvement in cofactor specificity ratio. Enzymatic characterization reveals that the substrate spectrum of *Sp*ADH2 could change significantly when utilizing different totally synthetic NCBs (tsNCBs). Furthermore, interaction analysis demonstrates critical roles of residues 43 and 290 in anchoring and release of *p*-BANA^+^. This study identified a natural ADH capable of utilizing totally synthetic NCBs, which has never been reported. Importantly, our results provide valuable ADH candidates for potential synthetic biology and industrial developments, and offer valuable guidance for identification and engineering ADHs toward utilizing NCBs as cofactors with improved catalytic performance.

**Supplementary Information:**

The online version contains supplementary material available at 10.1186/s40643-025-00870-z.

## Introduction

Alcohol dehydrogenases (ADHs), are crucial industrial biocatalysts that require stoichiometric amounts of cofactors to perform their catalytic functions (Headon and Walsh 1994). However, their application is often limited by the expensive natural cofactors, particularly nicotinamide cofactors, NAD(P)^+^/NAD(P)H, which comprised 80% of the cofactors used in oxidoreductases (Zachos et al. 2019). Over the years, numerous efforts have been carried out to relieve the cost of cofactors. The most applied approach involves cofactor regeneration, particularly through enzymatic methods (Dai et al. 2023, 2024; Ma et al. 2023; Sun et al. 2017), although these methods introduce unwanted by-products and cosubstrates. Other cofactor regeneration techniques, such as chemical, electrochemical, and photochemical methods suffer from drawbacks such as environmental pollution, low atom utilization, and reliance on photosensitizers or specialized media (Mordhorst and Andexer 2020).

To simplify the catalytic process of ADHs and reduce costs, researcher have dedicated to seek low-cost alternatives to natural nicotinamide cofactors. These alternatives, known as nicotinamide cofactor biomimetics (NCBs), have been developed over the past few decades. The first NCBs was synthesized in 1936 to give insights into the chemical properties of cofactors (Karrer and Stare 1936), and were later explored as substitutes for natural cofactors. Semi-synthetic NCBs (ssNCBs), such as nicotinamide mononucleotide (NMN) and nicotinamide cytosine dinucleotide (NCD), resemble natural cofactors in structure. NMN, for example, is an intermediate in the biosynthesis of NAD (Poddar et al. 2019) and has been applied in glucose-6-phosphate dehydrogenase as a glucose biosensor (Meng et al. 2023). NCD has been utilized in bio-orthogonal systems, with an intracellular synthetic pathway for NCD synthesis being developed through NCD synthetase (Wang et al. 2021). For totally synthetic NCBs (tsNCBs), they were considered as cost-efficient alternatives for natural cofactors (Guarneri et al. 2019). A thermostable ene-reductase *FOY*E-1 was reported to utilize BNAH as cofactor with 50-fold reduced cost (Tischler et al. 2020). In 2013, simple chemical synthesis routes were introduced, yielding a series of tsNCBs that retain only the nicotinamide moiety of NAD(P)H (Paul et al. 2013). They offered the potential for better catalytic efficiency compared with natural cofactors (Knaus et al. 2016). Further advancements have expanded the range of ssNCBs in the following decade (Falcone et al. 2019; Gu et al. 2025; Li et al. 2021; Nowak et al. 2017).

Much of the research in this area has been focused on improving in situ cofactor regeneration (Kara et al. 2014; Zhang and Hollmann 2018), biorthogonal screening (Liu et al. 2022; Richardson et al. 2020) as well as enhancing the activity (Löw et al. 2016; Qi et al. 2017). Unfortunately, the application of tsNCBs in ADH catalysis has been hold up by the scarcity of flavin-free oxidoreductases reported to utilize these cofactors. Instead, the majority findings focused on old yellow enzymes (Nowak et al. 2017; Paul et al. 2013). A NADH oxidase was explored for NCB regeneration, generating 3-carbamoyl-1-methylpyridin-1-ium (MNA^+^) and 1-benzyl-3-carbamoylpyridin-1-ium (BNA^+^) (Nowak et al. 2015). Some enzyme-NCB combinations outperformed natural combination (Geddes et al. 2016; Knaus et al. 2016). Some even displayed altered substrate spectrum (Löw et al. 2016), broadened the cofactor specificity (Campbell et al. 2010) or shifted redox reaction equilibria (Aspacio et al. 2024). Furthermore, certain ene-reductase showed remarkable asymmetric reduction of C = C bonds and carbon rings, demonstrating excellent conversion and enantioselectivity (Scholtissek et al. 2017; Tan et al. 2022).

Despite these advances, the identification of flavin-free oxidoreductases capable of utilizing tsNCBs has remained rare. Glucose dehydrogenase from *Sulfolobus solfataricus* was the first flavin-free oxidoreductases to accept tsNCBs (Nowak et al. 2017), and subsequent efforts focused on enhancing its activity and broadening its cofactor spectrum (Huang et al. 2019; Zachos et al. 2022; Zhou et al. 2023) have been reported. The ongoing search for oxidoreductases accepting tsNCBs is a new endeavor, but remains a significant area of research. However, to date, no breakthrough has been made regarding ADH capable of utilizing tsNCBs. Previously, an alcohol dehydrogenase from horse liver (HLADH) was suggested to accept tsNCBs (Jones and Taylor 1973; Lo and Fish 2002), but was later suspected as background activity which could only be detected in crude enzyme (Josa-Culleré et al. 2019), leading to debates about its true capability to utilize NCBs. Consequently, no ADH has been identified that can utilize tsNCBs, and an ADH from *Rhodococcus ruber* DSM 44541 has even been used as negative control in studies of NCBs utilization (Rocha et al. 2024).

*Sphingobium* sp. SYK-6 was first isolated from waste water of a paper mill in Japan, 1987, and was applied in lignin degradation (Katayama et al. 1987). An aldehyde dehydrogenase *Sp*ALDH2 from *Sphingobium* sp. SYK-6 was found to process broad substrate spectrum (Chen et al. 2024) and was essential in the metabolism of syringaldehyde (Kamimura et al. 2017). In this study, we identified an ADH from *Sphingobium* sp. SYK-6 (*Sp*ADH2), representing the first ADH capable of utilizing tsNCBs, particularly toward *para*-3-carbamoyl-1-(4-carboxybenzyl)pyridin-1-ium (*p*-BANA^+^). *Sp*ADH2 displayed distinct substrate spectrum when using *p*-BANA^+^ as a cofactor. Structural analysis of the enzyme-cofactor complex provides insights into molecular mechanism underlying the improved performance. Variant A290I exhibited a 10-fold increase in specific activity, while the double variant H43L/A290I achieved a remarkable 6750-fold increase in cofactor specificity ratio (CSR, ratio of catalytic efficiency of *p*-BANA^+^ to NAD^+^). Moreover, the cofactor spectrums of the variants were broadened and altered compared with WT. These findings, combined with structural insights gained, provide valuable opportunities for mining and identifying more ADHs favoring mimetic cofactors, and paves way for their potential application in industrial biocatalysis.

## Results and discussion

### Identification of alcohol dehydrogenases capable of utilizing NCBs

**Fig. 1:**
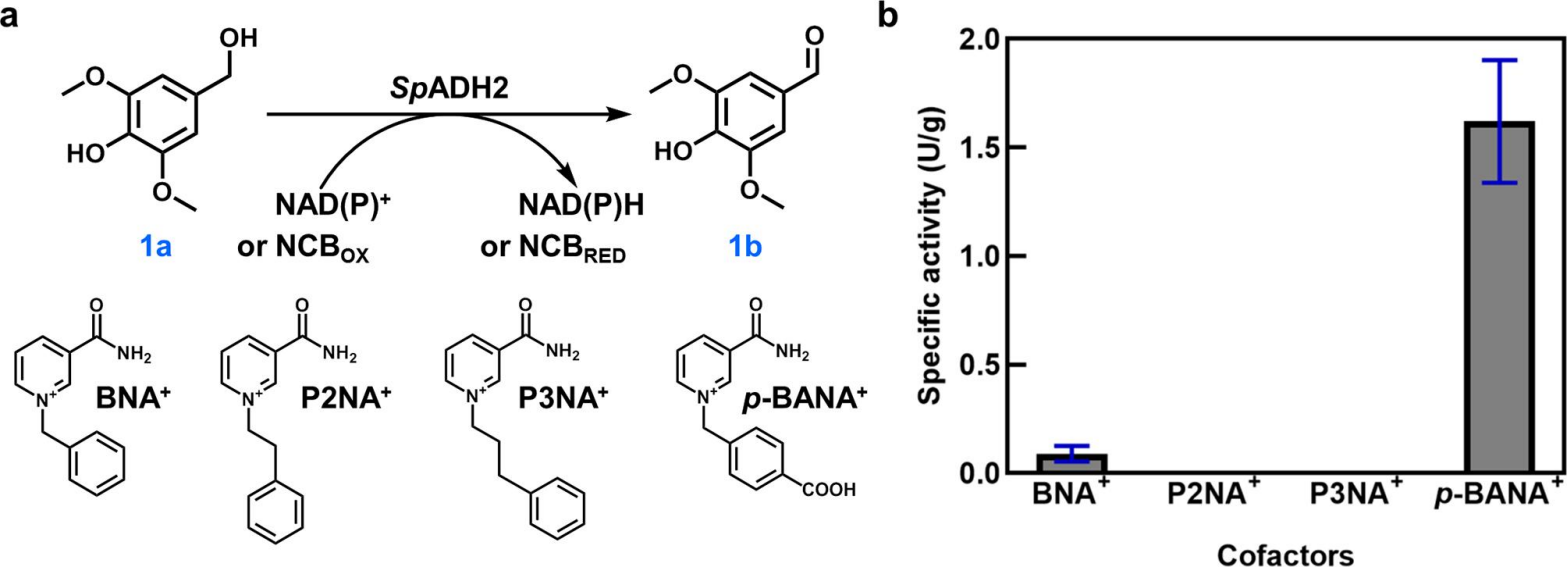
(**a**) Reaction scheme of *Sp*ADH2-catalyzed oxidation of syringyl alcohol. (**b**) Specific activity of *Sp*ADH2 toward four NCBs. The evaluation of NCBs usage by *Sp*ADH2 was carried out under following conditions: 50 mM PBS buffer (pH 7.0) and 30℃, with **1a** (1 mM) as substrate and 4 typical NCBs (10 mM) as cofactor. A total of 0.1 mg/mL enzyme was added to a 200 µL reaction mixture, and was analyzed by HPLC after 1 h reaction

*Sphingobium* sp. SYK-6 belongs to genus *Sphingomonas*, and a search in UniProtKB identified 19 ADHs from this strain, including 5 PQQ-dependent and the remaining NAD^+^/NADP^+^-dependent enzymes. Two sequences annotated as aryl-alcohol dehydrogenases (SLG_34580 and SLG_24930) (Table. S1) were selected for heterologous expression (Fig. S1) and named *Sp*ADH1 and *Sp*ADH2, respectively. Their ability to utilize NCBs was assessed by oxidation of syringyl alcohol (**1a**) to produce syringaldehyde (**1b**) (Fig. 1a). As shown in Fig. 1b, only *Sp*ADH2 effectively utilized NCBs, particularly *p*-BANA^+^ (bearing a *para*-carboxyl group), achieving a catalytic activity of 1.62 U/g. Appreciable activity was observed with BNA^+^, whereas 3-carbamoyl-1-phenethylpyridin-1-ium (P2NA^+^) and 3-carbamoyl-1-(3-phenylpropyl)pyridin-1-ium (P3NA^+^) could not be utilized by *Sp*ADH2.

### Exploration of cofactor and substrate spectra of *Sp*ADH2

**Fig. 2:**
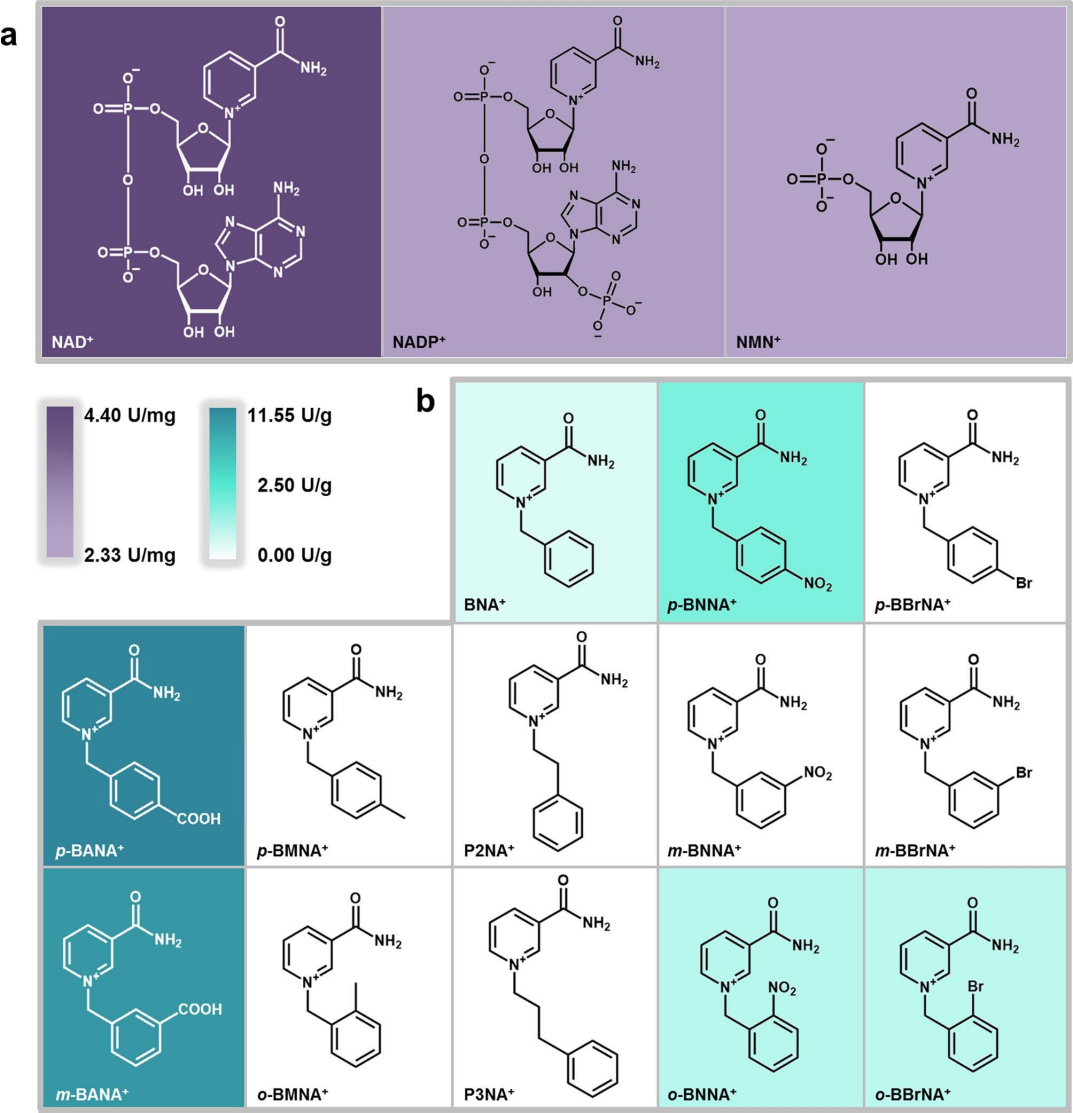
Cofactor spectrum of syringyl alcohol oxidation catalyzed by *Sp*ADH2. The darkness of the background color for each cofactor stands for the activity of *Sp*ADH2 utilizing various cofactors. (**a**) NAD(P) ^+^ and NMN^+^ are shown in purple background. (**b**) tsNCBs are shown in cyan background. The evaluation of cofactor spectrum of *Sp*ADH2 was carried out under following conditions: 50 mM PBS buffer (pH 7.5) and 30℃, with **1a** (5 mM) as substrate and different cofactors (7.5 mM) as cofactor. For NAD(P)^+^ and NMN^+^, 0.05 µg *Sp*ADH2 was added, and for tsNCBs, 5 µg *Sp*ADH2 was added. The results were analyzed by HPLC after 1 h reaction

Subsequently, the reaction condition of *Sp*ADH2 was optimized (Fig. S2-S3) and exploration of cofactor spectrum of *Sp*ADH2 was conducted (Fig. S4), including natural cofactors NAD^+^/NADP^+^, semi-synthetic cofactor NMN^+^, and a series of tsNCBs designed based on BNA^+^ scaffold. The results indicated that the optimum cofactor for *Sp*ADH2 was NAD^+^, achieving a specific activity of 4.40 U/mg (Fig. 2). In contrast, using NADP^+^ or NMN^+^ as cofactors resulted in a roughly 50% decrease in activity. Among all tsNCBs, *p*-BANA^+^ with a carboxyl group at the *para* position exhibited the highest activity of 11.55 U/g (a 7.1-fold increasement compared with preliminary assay), followed by *meta*-3-carbamoyl-1-(3-carboxybenzyl)pyridin-1-ium (*m*-BANA^+^) with a carboxyl group at *meta* position. In comparison, *meta*-3-carbamoyl-1-(3-nitrobenzyl)pyridin-1-ium (*m*-BNNA^+^) with a nitro group at *meta* position was not effectively utilized by *Sp*ADH2. However, when the nitro group was introduced at *ortho* or *para* positions, the activities were measured as 1.92 U/g and 2.25 U/g, respectively. Finally, results with bromine-substituted cofactors reveal that *Sp*ADH2 could only utilize cofactor with Br- at *ortho* position, achieving an activity of 1.3 U/g. No detectable activity was observed with methyl-substitutes, including *para*-3-carbamoyl-1-(4-methylbenzyl)pyridin-1-ium (*p*-BMNA^+^) and *ortho*-3-carbamoyl-1-(2-methylbenzyl)pyridin-1-ium (*o*-BMNA^+^), and also those with extended carbon-chain, such as P2NA^+^ and P3NA^+^. These results indicate that the cofactor recognition by *Sp*ADH2 is influenced by both the electronic properties of the substituents and their positions in cofactors.

**Fig. 3:**
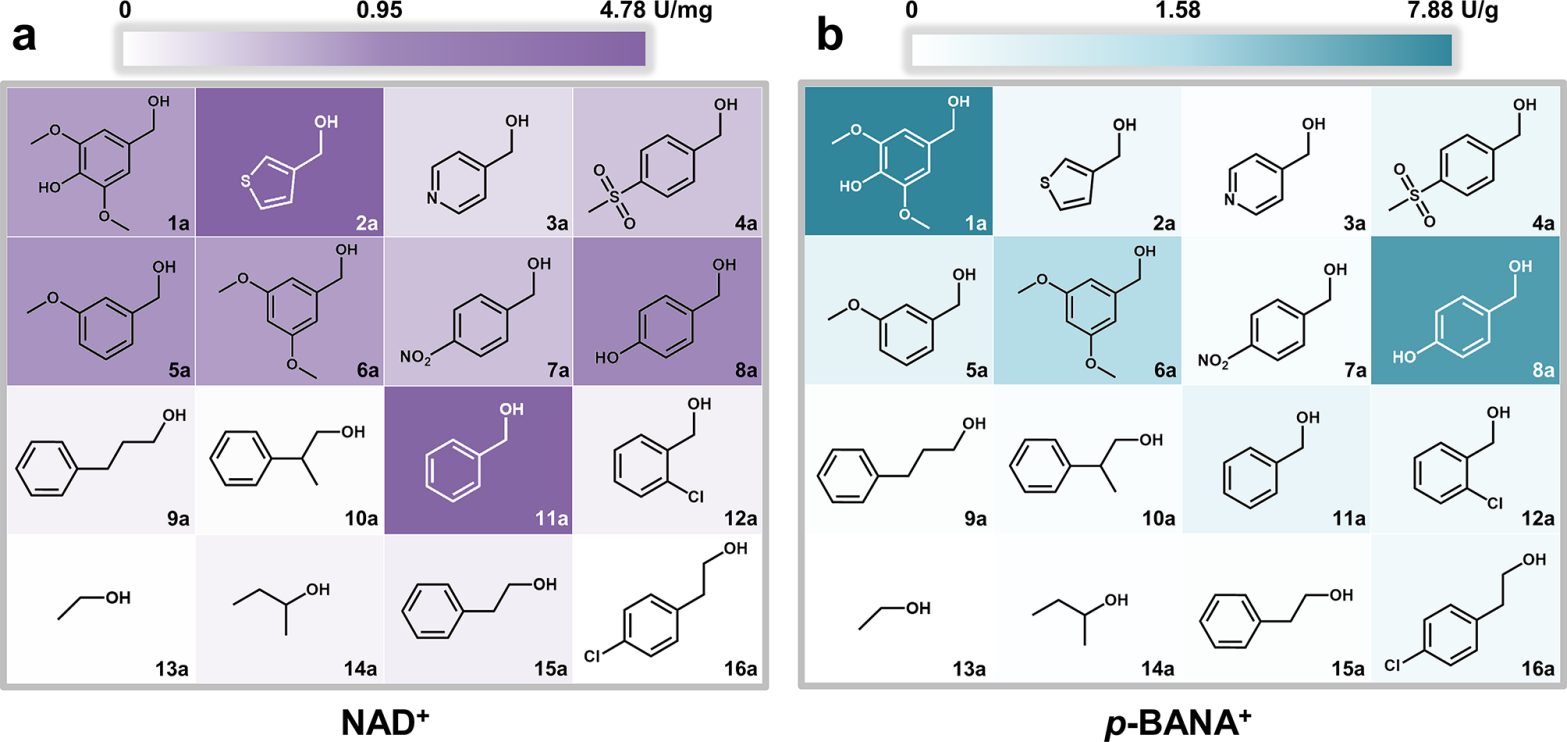
Substrate spectrum of *Sp*ADH2 utilizing (**a**) NAD^+^ and (**b**) *p*-BANA^+^. The darkness of the background color for each substrate stands for the activity of *Sp*ADH2 with NAD^+^ (purple) or *p*-BANA^+^ (cyan) as cofactor. The evaluation of substrate spectrum of *Sp*ADH2 was carried out under following conditions: 50 mM PBS buffer (pH 7.5) and 30℃, with **1a** (5 mM) as substrate. For NAD^+^(2 mM), 0.05 µg *Sp*ADH2 was added, and for *p*-BANA^+^(7.5 mM), 5 µg *Sp*ADH2 was added. After 1 h, reactions were visualized by 0.2 mM florescent probes (2-[2-(1,4-dihydro-1-methyl-3-quinolinyl)ethenyl]-1,3,3-trimethyl-3*H*-indolium)

The substrate spectrum of *Sp*ADH2 was subsequently tested using NAD^+^ and *p*-BANA^+^ (the optimum tsNCB) (Fig. 3). Overall, the catalytic activity with NAD^+^ as the cofactor was significantly higher than that with *p*-BANA^+^. A key finding was that the introduction of different cofactors resulted in a shift in substrate preference. For instance, in the presence of NAD^+^, the optimal substrates for *Sp*ADH2 were 3-thiophenemethanol and benzyl alcohol, with specific activities of 4.78 U/mg and 4.71 U/mg, respectively (Fig. 3a). In contrast, when *p*-BANA^+^ was used as the cofactor, the optimal substrate was syringyl alcohol, followed by *para*-hydroxybenzyl alcohol (Fig. 3b). No significant preference was observed for 3-thiophenemethanol and benzyl alcohol. This suggests that, for ADH-catalyzed reactions following the bi-bi mechanism, improving catalytic efficiency requires a comprehensive consideration of the structural characteristics of the “enzyme-substrate-cofactor” ternary complex.

### Semi-rational engineering for enhanced activity toward *p*-BANA^+^

**Fig. 4:**
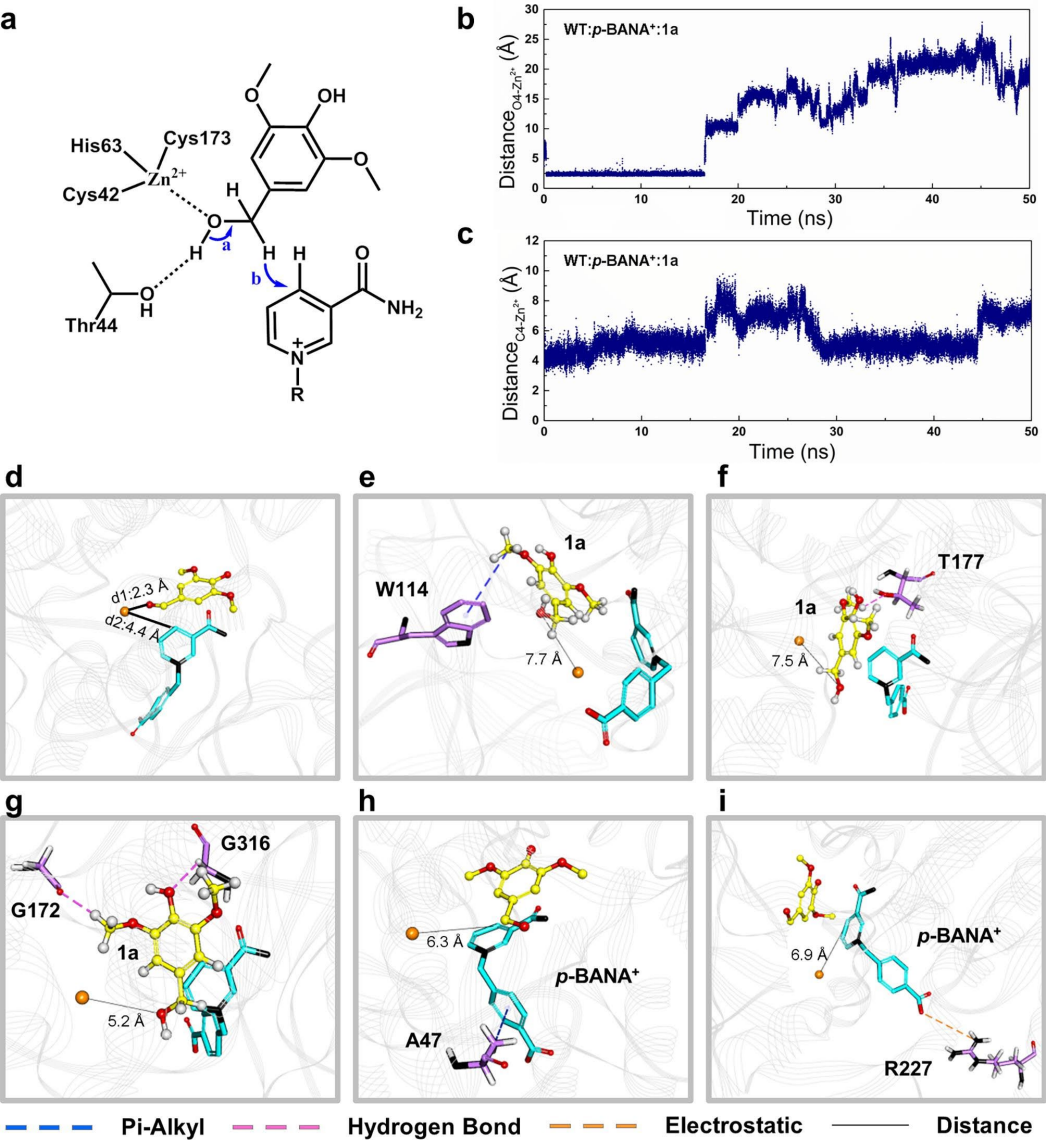
Reaction mechanism of medium-chain alcohol dehydrogenase *Sp*ADH2. (**a**) The proton (arrow a) and hydride (arrow b) transfer during the oxidation of **1a**. Catalytic Zinc ion is fixed by Cys42, His63, Cys173. (**b**) Changes in distance between Zn^2+^ and oxygen of the hydroxyl group of **1a** during 3 × 50 ns MD simulation. (**c**) Changes in distance between Zn^2+^ and C4 of the pyridinium group of *p*-BANA^+^ during 3 × 50 ns MD simulation. (**d**) Possible conformation of the *Sp*ADH2:*p*-BANA^+^:**1a** complex that correspond with the catalytic mechanism. Unfavorable interactions between **1a** and (**e**) W114, (**f**) T177, (**g**) G172 and G316. Unfavorable interactions between *p*-BANA^+^ and (**h**) A47, (**i**) R227

To enhance the catalytic activity of *Sp*ADH2 in the oxidation of **1a** using *p*-BANA^+^, a semi-rational engineering approach based on molecular dynamics simulations was employed. First, the three-dimensional structure of *Sp*ADH2 in complex with NAD^+^ was predicted using AlphaFold3. The substrate **1a** was then docked into the substrate-binding pocket. Figure 4a displays the overall structure. As a typical medium-chain alcohol dehydrogenase, the active site of *Sp*ADH2 contains a crucial catalytic Zn^2+^, which is coordinated by Cys42, His63, and Cys173. During the catalytic process, the hydroxyl oxygen of the key catalytic residue Thr44 transfers a proton to the hydroxyl group of **1a**, while the hydride is transferred to the C4 position of the pyridinium ring of the cofactor, ultimately yielding the product **1b** and the reduced cofactor.

To investigate the structural changes during the reaction process, the ternary complex obtained from docking was used as the initial structure for 50 ns molecular dynamics (MD) simulations. During the simulation, the distance between Zn^2+^ and the hydroxyl oxygen of **1a** (d1: O4-Zn^2+^), as well as the distance between Zn^2+^ and the C4 position of the pyridinium group of *p*-BANA^+^ (d2: C4-Zn^2+^), were monitored. Around 17 ns, both d1 and d2 showed a sudden increase in distance. Specifically, d1 increased from 2 Å to 10 Å (Fig. 4b). Representative conformations were selected for further analysis, revealing that the substrate **1a** was fixed by F109, C173 and G315 in favorable catalytic conformation (Fig. 4d and Fig. S5), but might rotate away when the methoxies groups form unwanted bonds. In those cases, W114 (Fig. 4e), T177 (Fig. 4f), G172 (Fig. 4g), and G316 (Fig. 4g) began to form new interactions such as π-alkyl interactions and hydrogen bond with the substrate, rotating it and pulling hydroxy group away from the catalytic center. Similarly, d2 increased from 4 Å to 8 Å (Fig. 4c), with A47 (Fig. 4h) and R227 (Fig. 4i) forming new π-alkyl and electrostatic interactions with *p*-BANA^+^, further pulling the cofactor away. Based on these findings, saturation mutagenesis libraries were created targeting these six positions to prevent the formation of these unfavorable new interactions, thereby ensuring a more stable binding between the substrate and cofactor at the active site of *Sp*ADH2.

**Fig. 5:**
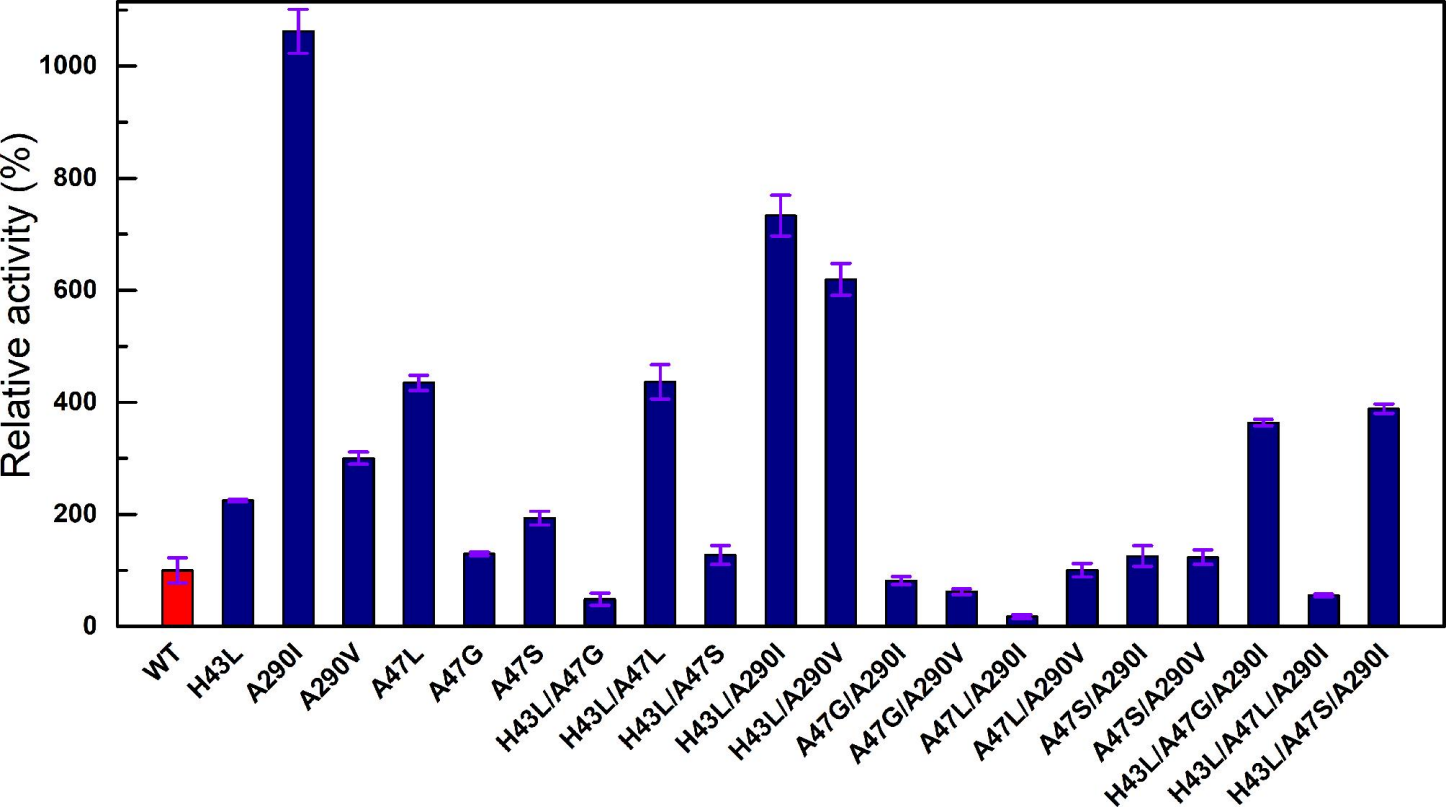
Relative activity of *Sp*ADH2 and its variants in oxidation of syringyl alcohol when utilizing *p*-BANA^+^ as cofactor

**Fig. 6:**
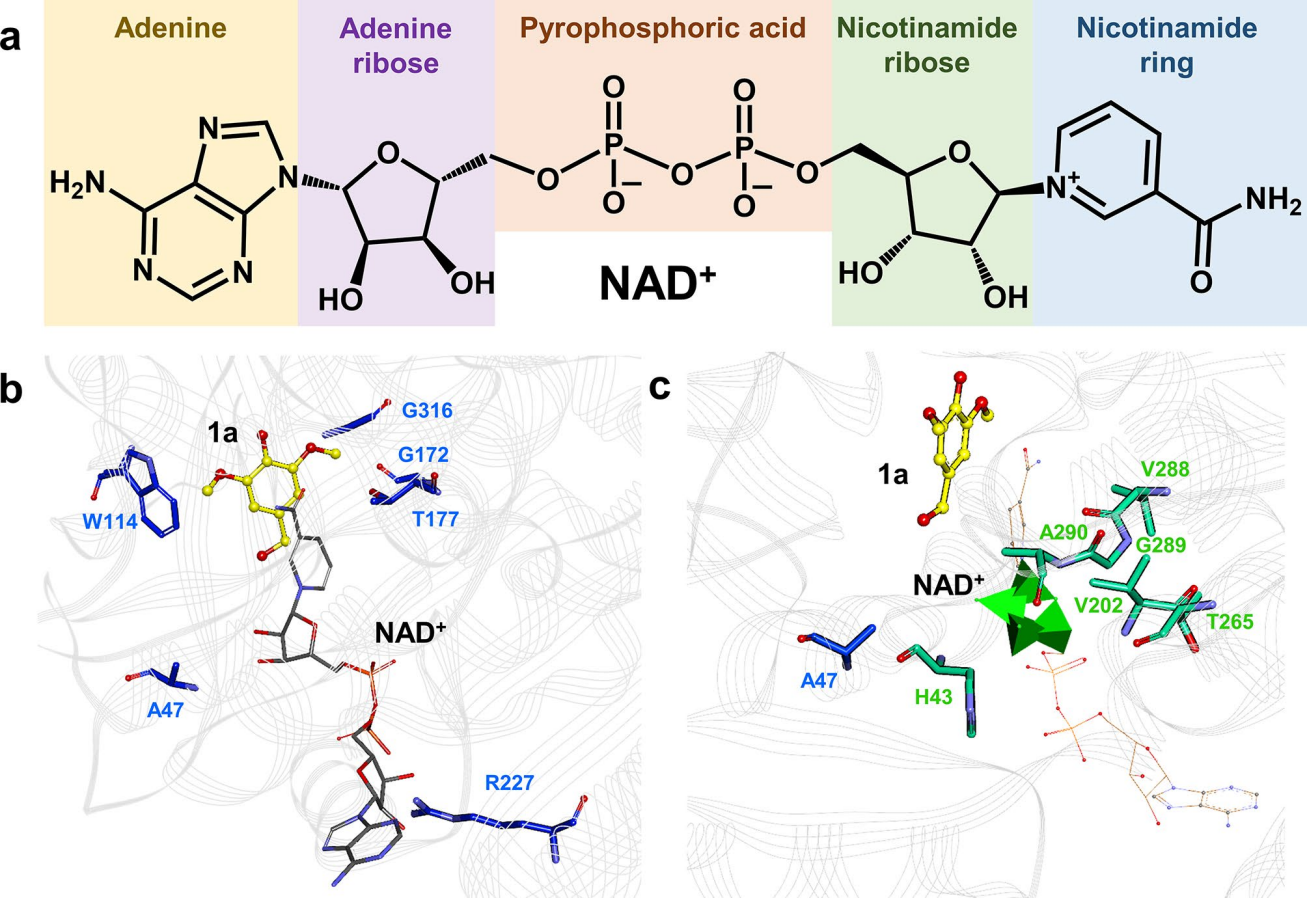
Identification of mutagenesis hotspots of *Sp*ADH2. (**a**) Structural formula of NAD^+^. (**b**) Mutation sites in the first round of mutagenesis identified by MD analysis, represented as blue sticks. (**c**) Mutation sites in second round of mutagenesis located within 5 Å around nicotinamide riboside of NAD^+^, represented as green sticks. The nicotinamide ribose moiety is presented in green triangular pyramids

In the first round of screening, saturation mutagenesis was constructed for six positions (W114, T177, G172, G316, A47, and R227). Only site A47 resulted beneficial variants, with A47L, A47G, and A47S exhibiting 4.3-, 1.3- and 1.8-fold higher activities than WT (Fig. 5). Upon revisiting the 3D structure of *Sp*ADH2-NAD^+^, it was found that W114 and G172 were located outside the 5 Å range of NAD^+^, T177 and G316 were near the nicotinamide ring, R227 was close to the adenosine ribose, and A47 was adjacent to the nicotinamide ribose (Fig. 6a and b). Therefore, in the second round of mutagenesis, six additional sites within the 5 Å range of the cofactor ribose (H43, V202, T265, V288, G289, and A290) were selected for saturation mutagenesis screening (Fig. 6c and Fig. S6). Three beneficial mutations were identified. Variants H43L and A290V exhibiting 2- and 3-fold increases in activity. Remarkably, A290I showed a 10-fold increase, reaching 83.64 U/g (Fig. 5). Subsequently, combinatorial mutagenesis was performed, resulting in 11 double variants and 3 triple variants. However, none of the combinatorial variants displayed significant synergistic effects, with their activities lower than that of A290I. The best combination mutant H43L/A290I exhibited an activity of 70 U/g, which is 7-fold higher than that of the WT.

### Kinetic assays of *Sp*ADH2 and its variants

Subsequently, the apparent kinetic parameters for substrate and cofactor were determined for WT, A290I, H43L, and H43L/A290I. In the substrate kinetic measurements, the presence of substrate inhibition was observed, regardless of NAD^+^ or *p*-BANA^+^ was used as the cofactor (Fig. S7-S8). As shown in Table 1, when NAD^+^ was used, all variants displayed reduced *k*_cat_ values compared with WT. Notably, variant H43L/A290I showed a 117-fold increase in *K*_m_ value, indicating significantly lower affinity toward NAD^+^. *K*_i_ of different variants vary greatly. H43L/A290I demonstrated an up to 9-fold improvement in substrate inhibition, and no substrate inhibition was observed with A290I. Mutation at site 290 might facilitate the substrate entry. In contrast, when *p*-BANA^+^ was employed as the cofactor, the *k*_cat_ values of H43L and A290I were increased by 2- and 10-fold, respectively. Remarkably, H43L/A290I showed a 6-fold increase in *k*_cat_, with 30% reduce in *K*_m_ value, resulting in a strikingly enhanced CSR (ratio of catalytic efficiency of *p*-BANA^+^ to NAD^+^) for 6750-fold.

Cofactor kinetic analysis reveals that both WT and variants exhibit high affinity for natural cofactor NAD^+^ (Fig. S9-S10). As shown in Table 2, variant A290I exhibited a 1.5-fold higher catalytic efficiency (*k*_cat_/*K*_m_ = 3144 min⁻¹∙mM⁻¹) than that of WT, whereas variant H43L/A290I showed *k*_cat_/*K*_m_ of merely 34.5 min⁻¹∙mM⁻¹, approximately 10% of A290I. Using *p*-BANA^+^ as the cofactor, apparent inhibitory effect was observed, evidenced by *K*_i_ values of 5.4−18.5 mM. Compared with NAD^+^, *p*-BANA^+^ is smaller in size, rendering it being more flexible in the cofactor binding site, which may occupy the cofactor binding domain in a non-catalytic conformation, resulting in cofactor inhibition. The catalytic efficiency of A290I (*k*_cat_/*K*_m_ = 0.93 min⁻¹∙mM⁻¹) was enhanced by 10-fold compared with WT, primarily due to 7.9-fold increase in *k*_cat_ value. Notably, the extremely low affinity and catalytic efficiency of H43L/A290I toward NAD^+^ resulted in relative specificity (RS, CSR ratio of variant to WT) value approaching 500. These results collectively demonstrate that variant H43L/A290I exhibits a markedly enhanced preference for *p*-BANA^+^ as a cofactor.

**Table 1:** Substrate kinetic parameters of *Sp*ADH2 and its variants

Cofactor	Enzyme	*K*_m_ (mM)	*k*_cat_ (min^− 1^)	*K*_i_ (mM)	*k*_cat_/*K*_m_ (min^− 1^∙mM^− 1^)	Cofactor specificity ratio	Relative specificity
NAD^+^	WT	0.017 ± 0.001	134 ± 2.9	5.5 ± 0.6	7882		
	H43L	0.339 ± 0.020	102 ± 2.3	10.5 ± 0.7	301		
	A290I	0.010 ± 0.004	41.4 ± 1.4	--	4140		
	H43L/A290I	2.00 ± 0.37	18.9 ± 0.2	48 ± 19	9.45		
*p*-BANA^+^	WT	9.4 ± 2.8	1.1 ± 0.3	7.4 ± 2.4	0.12	1.52 × 10^− 5^	
	H43L	8.8 ± 4.2	2.5 ± 0.9	7.7 ± 3.5	0.28	9.30 × 10^− 4^	61.2
	A290I	9.0 ± 4.2	11 ± 4.1	7.0 ± 3.6	1.22	2.95 × 10^− 4^	19.4
	H43L/A290I	7.0 ± 2.2	6.8 ± 1.6	8.0 ± 2.8	0.97	0.1026	6750

Cofactor specificity ratio (CSR): ratio of catalytic efficiency of *p*-BANA^+^ to NAD^+^. Relative specificity (RS): CSR ratio of variant to WT.

**Table 2:** Cofactor kinetic parameters of *Sp*ADH2 and its variants

Cofactor	Enzyme	*K*_m_ (mM)	*k*_cat_ (min^− 1^)	*K*_i_ (mM)	*k*_cat_/*K*_m_ (min^− 1^∙mM^− 1^)	Cofactor specificity ratio	Relative specificity
NAD^+^	WT	0.028 ± 0.003	58.7 ± 1.5	--	2096		
	H43L	0.099 ± 0.008	57.8 ± 1.2	--	584		
	A290I	0.009 ± 0.001	28.3 ± 0.6	--	3144		
	H43L/A290I	0.365 ± 0.025	12.6 ± 0.2	--	34.5		
*p*-BANA^+^	WT	20 ± 7.3	1.6 ± 0.5	12 ± 4.7	0.08	3.81 × 10^− 5^	
	H43L	28 ± 13	5.1 ± 2.0	5.4 ± 2.5	0.18	3.08 × 10^− 4^	8.08
	A290I	14 ± 3.8	13 ± 2.5	18 ± 5.8	0.93	2.96 × 10^− 4^	7.77
	H43L/A290I	13 ± 2.7	8.7 ± 1.3	15 ± 3.5	0.67	1.94 × 10^− 2^	509

Cofactor specificity ratio (CSR): ratio of catalytic efficiency of *p*-BANA^+^ to NAD^+^. Relative specificity (RS): CSR ratio of variant to WT.

### Molecular docking and interaction analysis of *Sp*ADH2 WT and variants with *p*-BANA^+^

**Fig. 7:**
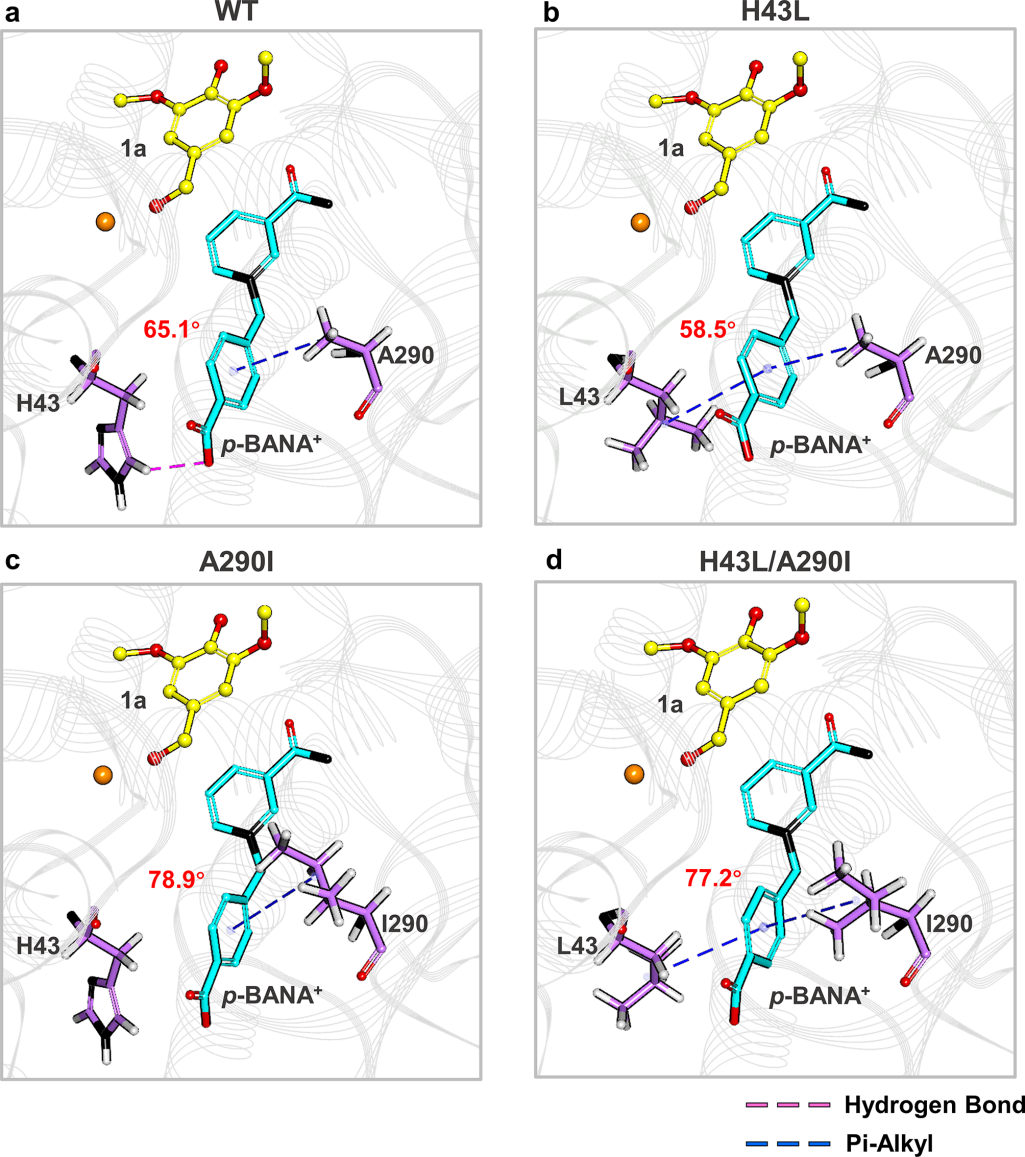
Cofactor interactions with residue 43 of *Sp*ADH2 and its variants. Cofactor *p*-BANA^+^, residue 43 and 290 are colored by elements and presented in stick. Substrate **1a** is colored by elements and presented in ball and stick. Cofactor interactions between residue 43 and 290 are demonstrated as dotted lines, with hydrogen bond in pink and π-alkyl interaction in blue. Dihedral angles between nicotinamide ring and phenyl group of *p*-BANA^+^ are labeled in red. (**a**) WT. (**b**) H43L. (**c**) A290I. (**d**) H43L/A290I

Molecular docking-based ligand in situ energy minimization was employed to analyze the structural distinctions between *Sp*ADH2 WT and its variants. Conformations of the enzyme-cofactor-substrate complexes reveal significant changes in the interactions between site 43 and *p*-BANA^+^ (Fig. 7). For WT, histidine at site 43 forms a hydrogen bond with the carboxyl group of *p*-BANA^+^, which may hinder the release of the reduced cofactor after reaction. For H43L, upon mutation of histidine to leucine, the interaction between residue 43 and *p*-BANA^+^ changed from hydrogen bond to weaker π-alkyl interaction, which could be favorable for cofactor release. For A290I, the mutation did not affect the anchoring of *p*-BANA^+^ through π-alkyl interaction with site 290. However, with enlarged alkyl side chain of isoleucine, the dihedral angle between nicotinamide ring and phenyl group of *p*-BANA^+^ increased from 65.1° (WT) to 78.9°, resulting in loss of any observable interaction between site 43 and *p*-BANA^+^. For double variant H43L/A290I, leucine at site 43 was able to re-establish the π-alkyl interaction with *p*-BANA^+^, the same as in H43L. Above interaction analysis highlights the beneficial effects of mutations H43L and A290I in stabilizing cofactor through appropriate interactions. For nicotinamide cofactor-dependent oxidoreductases, it is essential to ensure that the cofactor is not only securely anchored, but also readily released.

### Cofactor spectrum of *Sp*ADH2 and its variants

**Fig. 8:**
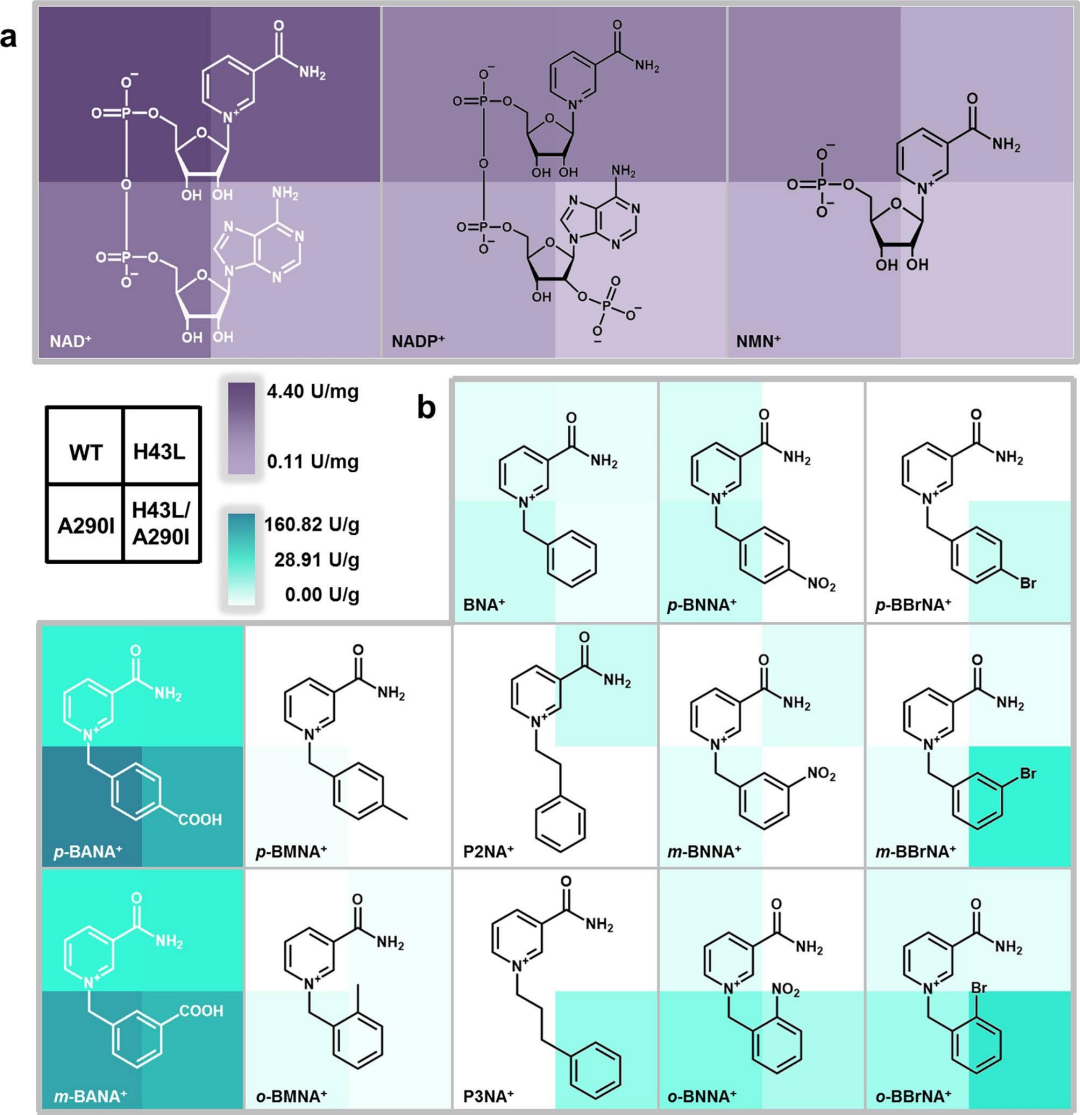
Cofactor spectrums of *Sp*ADH2 and its variants. The darkness of color for each cofactor stands for the activity. For each cofactor cube, top left: WT, top right: H43L, bottom left: A290I, bottom right: H43L/A290I. (**a**) Natural cofactors and NMN^+^ are shown in purple. (**b**) tsNCBs are shown in cyan. The evaluation of cofactor spectrum was carried out under following conditions: 50 mM PBS buffer (pH 7.5) and 30℃, with **1a** (5 mM) as substrate and different cofactors (7.5 mM) as cofactor. For NAD(P)^+^ and NMN^+^, 0.05 µg enzyme was added, and for tsNCBs, 5 µg enzyme was added. The results were analyzed by HPLC after 1 h reaction

Finally, the cofactor profile of WT and variants was determined. All variants displayed relatively lower catalytic activity when utilizing NAD^+^, NADP^+^ and NMN^+^(Fig. 8). For tsNCBs, cofactor specificity of the variants differed from each other. Apparently, all variants showed the highest activity toward *p*-BANA^+^ and *m*-BANA^+^, with A290I achieving prominent catalytic activities of 160.82 U/g and 118.11 U/g, respectively, followed by double variant H43L/A290I. In contrast, protein engineering did not result in a significant improvement toward utilizing of methyl-substituted cofactors, likely due to the electron-donating properties of the methyl group. H43L was the only variant exhibited activity with P2NA^+^, while variant H43L/A290I acquired capability of utilizing P3NA^+^, warranting further investigation. Additionally, H43L/A290I demonstrated the highest activity when utilizing a series of bromine (Br)-substituted cofactors, with activity following the order: *ortho* > *meta* > *para*. A290I was the only variant capable of utilizing cofactors with nitro substitutions at all three positions (*o*-, *m*-, and *p*-BNNA^+^), whereas variant H43L could only utilize the *meta*-substituted *m*-BNNA^+^. By engineering cofactor specificity, multiple NCBs could be regenerated by *Sp*ADH2 variants, which could serve as cofactor regeneration in biocatalytic reactions. For example, *Sp*ADH2 could regenerate reduced NCBs for reactions catalyzed by enoate reductase (*Ts*ER) (Nowak et al. 2017) or xenobiotic reductase A (XenA) (Knaus et al. 2016), to synthesize compounds such as ketoisophorone.

## Conclusion

NCBs, being cost-efficient and stable, are advantageous in industrial applications. This study successfully identified *Sp*ADH2 as a promising candidate for utilizing tsNCBs, particularly *p*-BANA^+^, for the oxidation of a series of aryl alcohols. Exploration of cofactor and substrate spectra of *Sp*ADH2 reveals the key role of cofactor structure in influencing enzymatic activity and substrate specificity. Employing semi-rational engineering guided by MD simulations, significant improvements in catalytic performance were achieved, particularly with variants A290I and H43L/A290I. These variants exhibited enhanced activity and cofactor specificity toward tsNCBs, especially *p*-BANA^+^ and *m*-BANA^+^, providing new insights into the molecular basis of cofactor recognition in alcohol dehydrogenases. Moreover, the observed shift in cofactor preference among variants expands the types of NCBs available for oxidoreductases to produce high-value compounds. This diversity enables their application across different cofactor regeneration systems with atomic economy. The results suggest that tailor-made engineering can lead to highly efficient tsNCBs-utilizing ADHs variants for applications in synthetic biology and industrial catalysis, paving the way for the development of cost-effective and versatile biocatalysts. Further investigation into detailed mechanisms of cofactor recognition and “enzyme-substrate-cofactor” catalytic complex will be essential for future *de novo* design and industrial practice.

## Materials and methods

### Regents, strains and plasmids

All substrates, standard products and reagents used for NCBs synthesis were purchased from Macklin Inc. (Macklin, Shanghai, China). Other biochemicals and medium components were purchased from China National Medicines Corporation Ltd. (Sinopharm, Wuhan, China). The sequence of *Sp*ADH1 and *Sp*ADH2 were commercially synthesized (Yixin, Wuxi, China). Oligonucleotide primers for PCR were synthesized by Tianlin Biotechnology Wuxi Co. Ltd.

All plasmids were preserved in our laboratory and used for expressing in *Escherichia. coli* BL21 (DE3). Genes encoding *Sp*ADH1 and *Sp*ADH2 were codon optimized for *E. coli*, commercially synthesized (Yixin, Wuxi, China) in pET28a plasmid. The optimized sequences are shown in Table S1.

### Synthesis of oxidized tsNCBs

Based on previously developed procedures (Falcone et al. 2019; Gu et al. 2025; Nowak et al. 2017; Paul et al. 2013), nicotinamide with a final concentration of 1 mol/L was added to a round-bottom flask (100 mL), and 40 mL of acetonitrile was used as a solvent, and a magnetic stirrer was used to stir evenly into a turbid liquid. Bromide with a final concentration of 1 mol/L was added to round-bottom flasks, and the reaction was carried out for 16 h at 50℃. Diethyl ether of 50 mL was added to the flask in batches, and the precipitate obtained was washed with ether, DCM, hexane and ether sequentially. Then the precipitate was filtered using a Brinell funnel, and air-dried in a surface dish to obtain products in powder.

### Recombinant strains and gene expression

*E. coli* was used as the host for cloning and expression of target genes, and was cultured in the LB media supplemented with 50 µg/mL kanamycin. Target plasmid was transferred to *E. coli* competent cells. Then the recombinant strain was cultured at 37°C and 180 rpm, and was induced by 1 mmol·L^− 1^ IPTG for 20 h at 16°C and 180 rpm. Cells were collected and resuspended in 50 mmol·L^− 1^ PBS buffer (pH 7.5), disrupted by sonicator, centrifuged at 4°C for 20 min. The supernatant was collected, and the protein expression was analyzed by SDS-PAGE. The remaining crude enzyme solution was used for enzyme purification.

Target enzymes were purified using 10 mL Ni-NTA affinity chromatography columns, and his-tagged enzymes were eluted by elution buffer (50 mM PBS, 500 mM imidazole, pH 7.5). The eluents were then desalted with 10 mL HiTrap desalting column on ӒKTA pure™ (Cytiva, Shanghai). Purified enzymes were confirmed by SDS-PAGE, and the protein concentrations were estimated by Nanodrop 2000c (Thermo Scientific Inc, USA).

### HPLC analysis of specific activity

Reaction samples were analyzed using Agilent 1260 Infinity II LC system under reversed phase condition. Column: Agilent C18 column. Analytical conditions for **1a** and **1b**: mobile phase: 50% water: 50% acetonitrile. The retention time for standard product **1b** was 3.27 min, under flow rate of 1 mL·min^− 1^and temperature of 30°C (Fig. S4).

Specific activity of *Sp*ADH2 in cofactor spectrum study was determined by HPLC analysis. PBS (pH 7.5) buffer was pre-heated at 30°C for 15 min. For NAD(P)^+^ and NMN^+^, 0.05 µg *Sp*ADH2 was added to PBS buffer (100 µL). For tsNCBs, 5 µg *Sp*ADH2 was added. A final concentration of 7.5 mM cofactor was added to the reaction mixture. Finally, a final concentration of 5 mM substrate was added to start the reaction. The reactions were conducted at 30°C.

One unit of enzyme activity is defined as the amount of enzyme required to form 1 µmol **1b** per minute at pH 7.5 and 30°C. Same reaction without cofactor was used as a blank control.

### Spectrophotometric analysis of specific activity

Specific activity of *Sp*ADH2 in substrate spectrum study was measured spectrophotometrically. PBS (pH 7.5) buffer was pre-heated at 30°C for 15 min. For NAD^+^, 0.05 µg *Sp*ADH2 was added to the PBS buffer (100 µL), and a final concentration of 2 mM NAD^+^ was added. For tsNCBs cofactor *p*-BANA^+^, 5 µg *Sp*ADH2 and a final concentration of 7.5 mM *p*-BANA^+^ was added. The mixture was carried out in a 96-well microplate, and a final concentration of 5 mM substrate was added to start the reaction. The microplate was incubated in 30°C for 1 h. Florescent probes (2-[2-(1,4-dihydro-1-methyl-3-quinolinyl)ethenyl]-1,3,3-trimethyl-3*H*-indolium) in a final concentration of 0.2 mM (100 µL) was added (Scheme S1), and the microplate was placed in a microplate reader immediately for measurement under 537 nm (Fomin et al. 2016).

One unit of enzyme activity is defined as the amount of enzyme required to form 1 µmol of reduced florescent probes per minute at pH 7.5 and 30°C. Each reaction was repeated in triplet. Same reaction without substrate was used as a blank control. The molar absorbance coefficient (ε) was detected as 53,718 µM^− 1^·cm^− 1^ with NADH as the reduced cofactor and 60,801 µM^− 1^·cm^− 1^ with *p*-BANAH as the reduced cofactor.

### Construction of mutagenesis library and high-throughput screening

Saturation mutagenesis was carried out using NNK codon. Primers are listed in Table. S2. Double and triple variants were generated using primers in Table. S2.

In preliminary screening by spectrophotometric analysis, PBS (pH 7.5) buffer (50 µL) in a 96-well plate was pre-warmed at 30°C for 15 min, and a final concentration of 5 mM *p*-BANA^+^ and 1 mM substrate syringyl alcohol were added, and 50 µL of crude enzyme were add to each well to start the reaction, with WT as control. The reaction mixture was incubated at 30°C for 1 h, followed by addition of fluorescent probes in a final concentration of 0.2 mM, and was subjected to determination under 537 nm as described above.

### Kinetic analysis

Kinetic analysis of *Sp*ADH2 and its variants were carried out following Michaelis-Menten equation or Michaelis-Menten excess substrate inhibition equation. For of the substrate kinetic assays, concentrations of cofactors were fixed at 5 mM for NAD^+^ and 7.5 mM for *p*-BANA^+^. For cofactor kinetic assays, the concentration of **1a** was fixed at 5 mM.

Specific activity was determined spectrophotometrically as above except that gradient **1a** (0−20 mM) or cofactor (0−35 mM) concentrations were applied.

Cofactor specificity ratio (CSR) represents the ratio of catalytic efficiency of *p*-BANA^+^ to NAD^+^ for each enzyme, and relative specificity (RS) stands for the CSR ratio of variants to WT (King et al. 2020).

## Electronic supplementary material

Below is the link to the electronic supplementary material.

Supplementary Material 1

Supplementary Material 2

Supplementary Material 3

## Data Availability

All data generated or analyzed during this study are included in this published article (and its supplementary information files).

## Abbreviations

NCBs: nicotinamide cofactor biomimetics
*Sp*ADH2: alcohol dehydrogenase from *Sphingobium* sp. SYK-6
ssNCBs: semi-synthetic nicotinamide cofactor biomimetics
tsNCBs: totally synthetic nicotinamide cofactor biomimetics
CSR: cofactor specificity ratio
RS: relative specificity
