# Supplementary material for: Engineering a newly identified alcohol dehydrogenase from Sphingobium Sp. for efficient utilization of nicotinamide cofactors biomimetics
Source: Bioresour Bioprocess. 2025 May 5;12(1):41. doi: 10.1186/s40643-025-00870-z (PMC12052742; doi:10.1186/s40643-025-00870-z)
Supplement: Supplementary file 1 — Supplementary Material 1 [file 40643_2025_870_MOESM1_ESM.docx]

**Engineering a Newly Identified Alcohol Dehydrogenase from *Sphingobium* sp. for Efficient Utilization of Nicotinamide Cofactors Biomimetics**

**Yichun Zhu^1^, Jieyu Zhou^1^*****, Xiangyuan Gu^1^, Huiru Wang^1^, Hao Han^1^, Ye Ni^1^***

*^1^ Key laboratory of industrial Biotechnology, Ministry of Education, School of Biotechnology, Jiangnan University, Wuxi, 214122, Jiangsu, China*

contents

[Molecular docking and MD simulations 2](#_Toc191912263)

[Table S1. Selected alcohol dehydrogenases from *Sphingobium* sp*.* SYK-6. 3](#_Toc191912264)

[Table S2. Primers for site saturation mutagenesis. 4](#_Toc191912265)

[Scheme S1. Reaction catalyzed by *Sp*ADH2. 5](#_Toc191912266)

[Figure S1. SDS-PAGE analysis of *Sp*ADH2. 6](#_Toc191912267)

[Figure S2. Optimal pH of *Sp*ADH2. 6](#_Toc191912268)

[Figure S3. Optimum temperature of *Sp*ADH2. 7](#_Toc191912269)

[Figure S4. HPLC chromatograms of standard 1b and reaction sample (blue/red= 1 mM 1b/sample of 24 h). 7](#_Toc191912270)

[Figure S5. Conformation of *Sp*ADH2:*p*-BANA^+^:1a complex that correspond with the catalytic mechanism. 8](#_Toc191912271)

[Figure S6. A47 (blue) and Residues selected for second round of mutagenesis (green) and their position around natural cofactor NAD^+^. 8](#_Toc191912272)

[Figure S7. Kinetic assays of *Sp*ADH2 and its mutants towards 1a while utilizing NAD^+^ according to Michaelis-Menten excess substrate inhibition equation. 9](#_Toc191912273)

[Figure S8. Kinetic assays of *Sp*ADH2 and its mutants towards 1a while utilizing *p*-BANA^+^ according to Michaelis-Menten excess substrate inhibition equation. 10](#_Toc191912274)

[Figure S9. Kinetic assays of *Sp*ADH2 and its mutants towards NAD^+^ while oxidizing 1a according to Michaelis-Menten equation. 11](#_Toc191912275)

[Figure S10. Kinetic assays of *Sp*ADH2 and its mutants towards *p*-BANA^+^ while oxidizing 1a according to Michaelis-Menten excess substrate inhibition equation. 12](#_Toc191912276)

[NMR 12](#_Toc191912277)

[References 22](#_Toc191912278)

# Molecular docking and MD simulations

Structure of *Sp*ADH2-NAD^+^ complex was predicted by AlphaFold3. Substrate **1a** and nonnatural cofactor *p*-BANA^+^ were docked into *Sp*ADH2 via Discovery Studio, and the “enzyme-cofactor-substrate” ternary complexes were obtained through *in situ* ligand minimization.

All atom MD simulations were carried out with GROMACS 2019.6 (Abraham et al. 2015). Protein-ligands complex systems were built using CPU version of AmberTools2023 (Case et al. 2023) molecular dynamics package. Each apo-protein was protonated at pH 7.5 by employing H++ webserver (Anandakrishnan et al. 2012). Amber ff19SB force field (Tian et al. 2020) was used to perform the MD simulations, and each system was solvated within a 10 Å buffer of TIP3P water molecules (Jorgensen et al. 1983). Explicit counterions (Na^+^ and Cl^-^) were added to neutralize overall charge of the systems. With AM1-BCC charge model (Jakalian et al. 2002) along with amber General Amber Force Field (GAFF) force field (Wang et al. 2004), charges and parameters for ligands were treated by Antechamber module. Force fields of zinc ion and its neighboring atoms (cutoff was set as 2.8 Å) were parameterized through “MCPB.py” modeling tool (Li and Merz 2016), using the HF/6-31G* level of theory. Seven atoms (C42.SG / H63.NE2 / C92.SG / C95.SG / C98.SG / C106.SG / C173.SG) were attached to zinc ion by coordinate bonds. After a proper setup, the whole system was fully minimized using 20000 steps of steepest descent and 10000 steps of conjugate gradient methods to remove poor contacts and relax the systems. After minimization, to achieve a uniform density after heating dynamics, the systems were maintained for 1 ns of density equilibration under NPT conditions at a target temperature of 303 K and target pressure of 1.0 atm using Berendsen pressure bath. Finally, a productive MD simulation under NPT ensemble was conducted for 50 ns for each enzyme system without any restriction. During all MD simulations, covalent bonds containing hydrogen were constrained and an integration step of 2 fs was used for each enzyme system. Trajectory file was written every 1 ps.

# Table S1. Selected alcohol dehydrogenases from *Sphingobium* sp*.* SYK-6.

| alcohol dehydrogenase | Source | Protein sequence |
| --- | --- | --- |
| *Sp*ADH1 | *Sphingobium* sp. SYK-6 | GGATCCATGCGTGCATTTGGGTACAACCGCGCTCACGACTTGGCGGATTTCGCTTTGCGCCTGCGTGATATGGCCGATCCGGTACCGGGGCCGGGAGACTTATTGGTTTCGGTTCGTGCGTTCGCCTTGAATCCCGTGGATTGCAAAATTCGTCGCACGCGCGATGGTGGAGCCGATGCGCCTGTGATCTTAGGGTGGGATGCCGCTGGAGTAGTGGAAGCCGTTGGGCCCGGAGTAACCGGTTTCACACCGGGCGACGCGGTTTTTTATGCTGGGGATATTACACGCCCAGGCTCCTATGCGACTTTACAAACGGTCGACCACCGTCTGGTTGCGCACAAACCCGCCTCGTTAGATTTTGCGGACGCGGCCGCACTTCCGCTGACTGCTCTGACTGCACATGAAGCAATGTTAGAGCGTGGGATTGCCTATGATGCTGAATCTATTGTTCTGGTGATCGGGGGCGCGGGAGGGGTAGGTAGTATGGCCGTGCAGTTGATGAAAGCCCTGACACCGGCACGTGTTATCGCCACAGCCTCCCGCCCTGAGAGCGTAGCTTGGGCTCGCGCTATGGGAGCCGATGATGTAATCGGTCGTTCATTAGCCGACGGCTTAGCGGCATTAGGTCTGGCACCCGGTTCCCTGCATGCGGTTTTTTCGACAACTGGAACGGATGCGGCATTACCTGTTATGCCGTCCCTTTTGCGTCCCTTCGGACACGTTATGGTTATTGATGATCCGGCAACGCTTGACATTAAACCCTTTAAGCAAAAAGCGCTGAGTGTTCATTGGGAATACATGTTTGCCCGCGCCATGTTCGGATGTGCACCTGAGCGCCAAGGGGCGACCTTAGCACGTATTGCGGCACTGGTTGAGGCCGGTCGCATTCGCACGACCGCCACGCGTCAGCTGCCTGCGACATTAGATAATTTACGCGCTGCGCATGCAGCCTTGGAAGCCGGAACTGGGATTGGGAAGACCGTGATGGTCTGGTCCCTCGAG |
| *Sp*ADH2 | *Sphingobium* sp. SYK-6 | GGATCCATGACAACAACAACGCGTGCAGCCATCTGCTATGGGGCTGAGAAACCTTTCTCGATTGAAGATGTCACGTTAGATGACCCCCGTGAAGATGAAGTTCTTGTGCGCATCCATGCTTGTGGGATTTGCCACACTGATATGGCTGCCCGTGATTCACAGATCCCAACGCCATTGCCTATGGTCCTTGGACATGAGGGGGCTGGCGTCGTTGAAAAAGTGGGGGCCGCTATCACACACGTTAAGCCGGGTGATCGTGTAATTATGAGCTTCAATTCGTGTGGTCACTGCCCATCGTGCGCAATTGATCGCCCCACGTACTGCTATAATTTCGTCCCCGAGAACTGGACTGGTAAGCGCGCAGACGGTTCCGCTACTGCGCACCAAGACGGTACGGACGTTAACGCGAATTTCTTTGGACAAAGTAGTTTCGCCGCCCACGCCTTGGCTCATGCACGTAATGTAGTCAAGGTTCCGGAATCAGCCGCAGCAATTCCTCTTGCCACTCTTGCTCCACTGGGGTGCGGCCTGATGACTGGAGCTGGAGCTGTCTTGCGTTCAATGAATGTTCGCGCCGGGATGCCTATCGCTATCTTCGGGTCGGGTGCTGTAGGCTTGGCAGCGGTCATGGCCGCGAAGATTGCCGGGGCCAACCCGATCATTGCCGTAGATGTCCACGACAATCGTTTGGCCTTAGCTCGTGAAATGGGGGCGACTCACTCCATTAATGGGCGCACCCAGAACGCAGTTGAAGAGATCCGCCGTATCAGTCCACAGGGACTTGCTTACGCTTTTGATACGACCGGCCTGAAATCGATCATTGAGCAAGCCTTTGGCTTACTGTTACCGCTGGGTGTCTTAGGGCTTGTTGGAGCGTCCGCTCCGACCGAGATGTTGAATTTCAACGAAAGTTCACTGATGGGAGGTGGGAAGCGTGTCATCGGAATTTTGGGTGGAGACTCTGATTTACAAACGTTCTTACCAGAATTGATTGAACATCATTTGGCTGGGCGTTTCCCGCATGAGCGTCTTATCCGCACGTTTCCTTTCTCACAGATTAATGAAGCCTTCCACGCGGGTGAGAGTGGAGAGGTCGTGAAACCTGTGTTGGTCATGGAGGATCTCGAG |
|  |  |  |
|  |  |  |

# Table S2. Primers for site saturation mutagenesis.

| Mutant | Forward primer | Reverse primer |
| --- | --- | --- |
| A47 | CACACTGATATGNNKGCCCGTGAT | TGAATCACGGGCMNNCATATCAGT |
| H43 | TGTGGGATTTGCNNKACTGATATG | AGCCATATCAGTMNNGCAAATCCC |
| W114 | TTCGTCCCCGAGAACNNKACTGGTAAGCGC | TGCGCGCTTACCAGTMNNGTTCTCGGGGAC |
| G172 | CTTGCTCCACTGNNKTGCGGCCTG | CATCAGGCCGCAMNNCAGTGGAGC |
| T177 | TGCGGCCTGATGNNKGGAGCTGGA | AGCTCCAGCTCCMNNCATCAGGCC |
| V202 | GGGTCGGGTGCTNNKGGCTTGGCA | CGCTGCCAAGCCMNNAGCACCCGA |
| R227 | GTCCACGACAATNNKTTGGCCTTA | AGCTAAGGCCAAMNNATTGTCGTG |
| T265 | TACGCTTTTGATNNKACCGGCCTG | TTTCAGGCCGGTMNNATCAAAAGC |
| V288 | GTCTTAGGGCTTNNKGGAGCGTCC | AGCGGACGCTCCMNNAAGCCCTAA |
| G289 | TTAGGGCTTGTTNNKGCGTCCGCT | CGGAGCGGACGCMNNAACAAGCCC |
| A290 | GGGCTTGTTGGANNKTCCGCTCCG | GGTCGGAGCGGAMNNTCCAACAAG |
| G316 | ATCGGAATTTTGNNKGGAGACTCT | ATCAGAGTCTCCMNNCAAAATTCC |
| H43L | TGTGGGATTTGCCTGACTGATATG | AGCCATATCAGTCAGGCAAATCCC |
| A47G | CACACTGATATGGGGGCCCGTGAT | TGAATCACGGGCCCCCATATCAGT |
| A47L | CACACTGATATGTTGGCCCGTGAT | TGAATCACGGGCCAACATATCAGT |
| A47S | CACACTGATATGTCGGCCCGTGAT | TGAATCACGGGCCGACATATCAGT |
| H43L/A47G | CTGACTGATATGGGGGCCCGTGAT | TGAATCACGGGCCCCCATATCAGT |
| H43L/A47L | CTGACTGATATGTTGGCCCGTGAT | TGAATCACGGGCCAACATATCAGT |
| H43L/A47S | CTGACTGATATGTCGGCCCGTGAT | TGAATCACGGGCCGACATATCAGT |


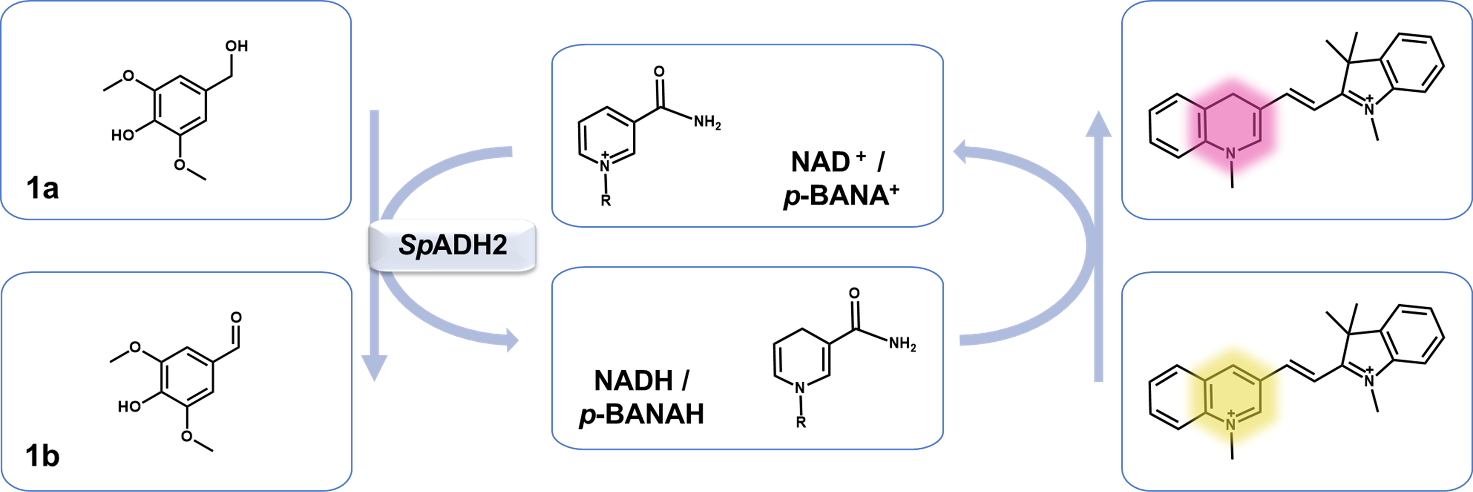


# Scheme S1. Reaction catalyzed by *Sp*ADH2.

**1a** is oxidized by *Sp*ADH2 to produce **1b**, companied by reduced cofactors which react with florescent probe and emit florescent pink color.

**
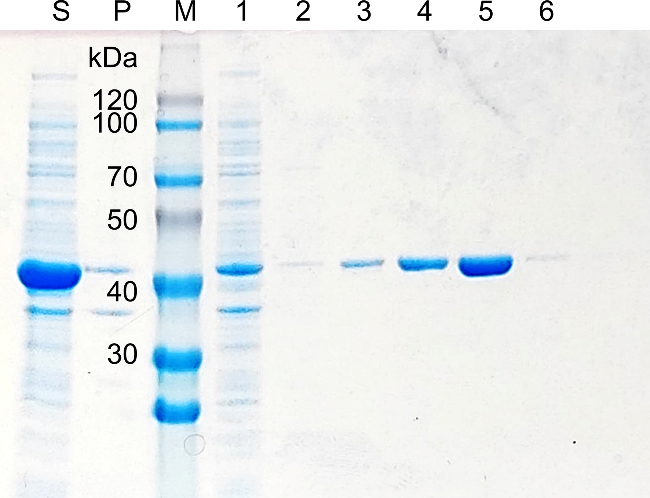
**

# Figure S1. SDS-PAGE analysis of *Sp*ADH2.

Lane S: supernatant, lane P: precipitation, lane M: marker, lane 1: flow through, lane 2: 50 mM imidazole elution, lane 3: 100 mM imidazole elution, lane 4: 150 mM imidazole elution, lane 5: 300 mM imidazole elution, lane 6: 500 mM imidazole elution. The target protein purified appears as a single band on SDS-PAGE.


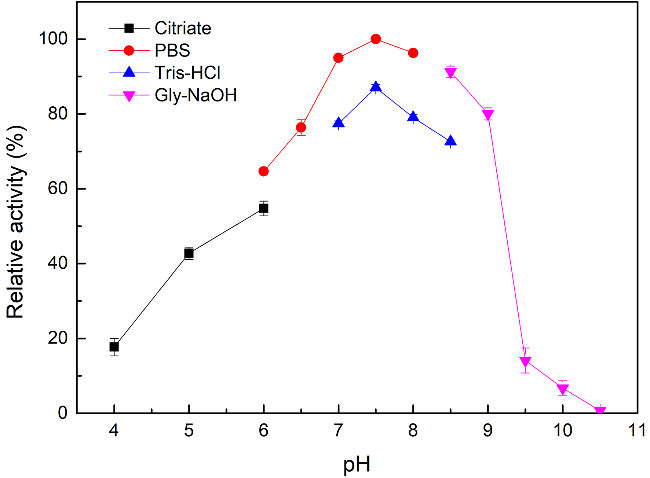


# Figure S2. Optimal pH of *Sp*ADH2.

Reaction conditions: c(**1a**) = 5 mM, c(NAD^+^) = 2 mM, c(*Sp*ADH2) = 0.1 mg/L, (purified enzyme), buffer: 50 mM, 30°C, triplicate measurements. Reaction volume = 100μL, detection volume = 200 μL, c(florescent probe) = 0.2 mM.


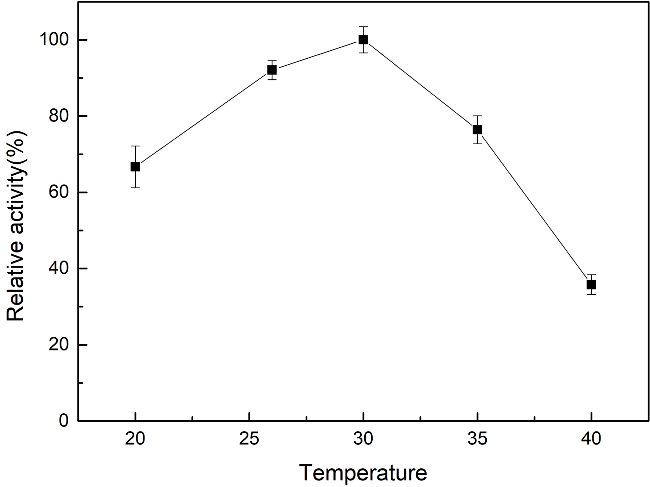


# Figure S3. Optimum temperature of *Sp*ADH2.

Reaction conditions: c(**1a**) = 5 mM, c(NAD^+^) = 2 mM, c(*Sp*ADH2) = 0.1 mg/L, (purified enzyme), buffer: 50 mM PBS (pH 7.5), triplicate measurements. Reaction volume = 100μL, detection volume = 200 μL, c(florescent probe) = 0.2 mM.


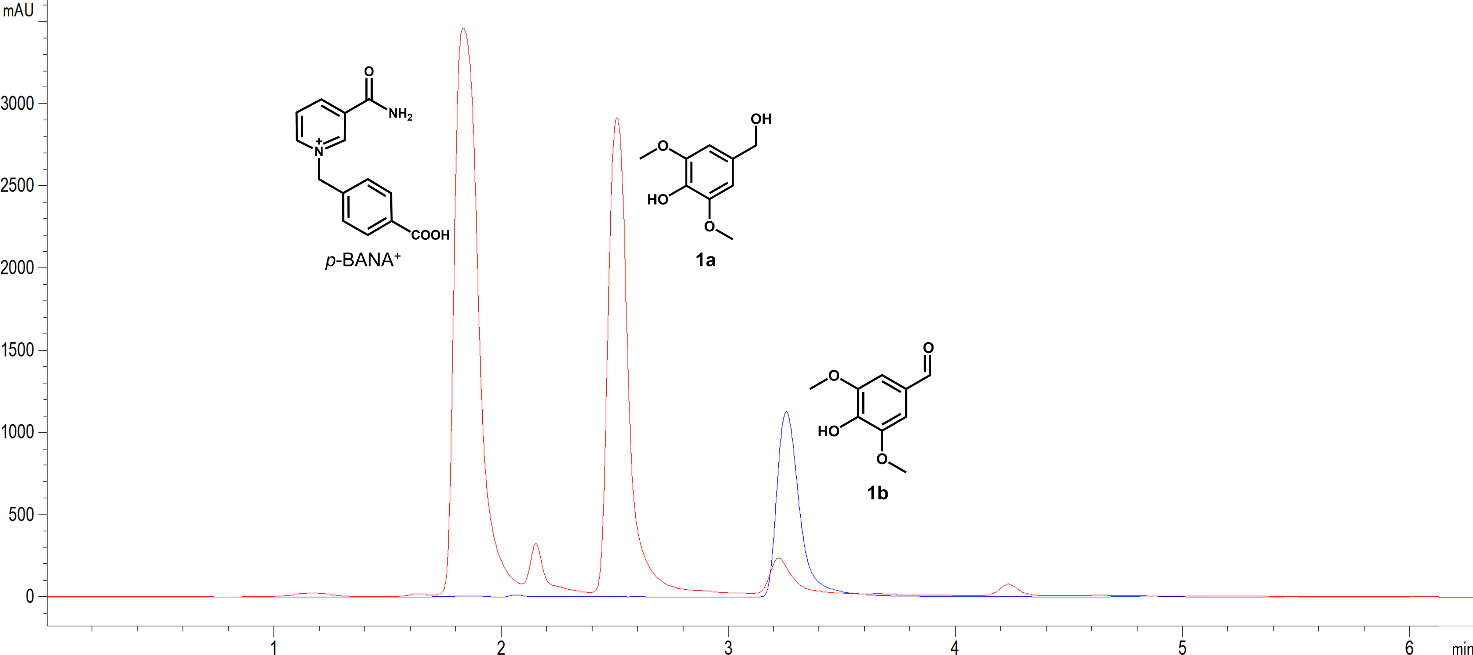


# Figure S4. HPLC chromatograms of standard 1b and reaction sample (blue/red= 1 mM 1b/sample of 24 h).

Reaction conditions: c(**1a**) = 5 mM, c(*p*-BANA^+^) = 7.5 mM, c(*Sp*ADH2) = 50 mg/L, (purified enzyme), buffer: 50 mM PBS (pH 7.5), 30°C, standing for 24 h.


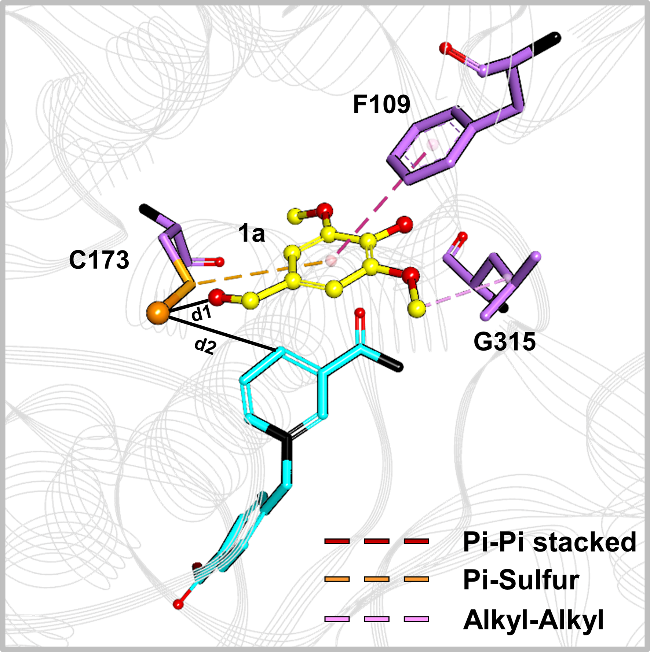


# Figure S5. Conformation of *Sp*ADH2:*p*-BANA^+^:1a complex that correspond with the catalytic mechanism.

Substrate **1a** is fixed by F109, C173 and G315 with π-π stacking, π-sulfur and alkyl-alkyl interactions.


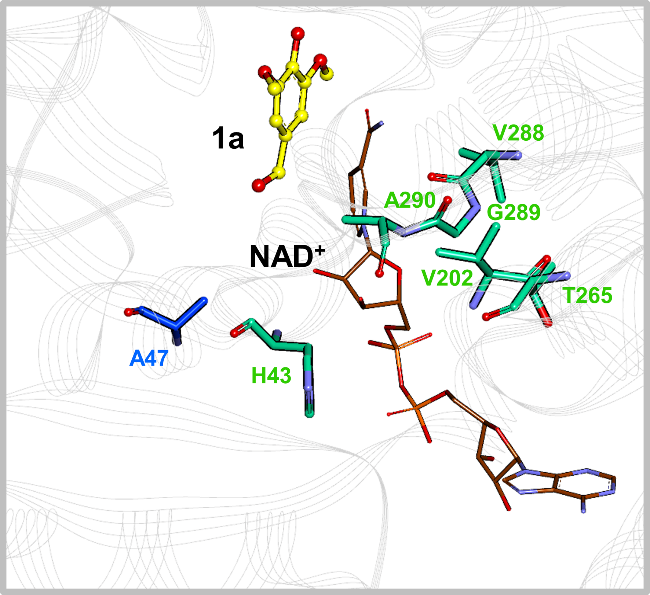


# Figure S6. A47 (blue) and Residues selected for second round of mutagenesis (green) and their position around natural cofactor NAD^+^.


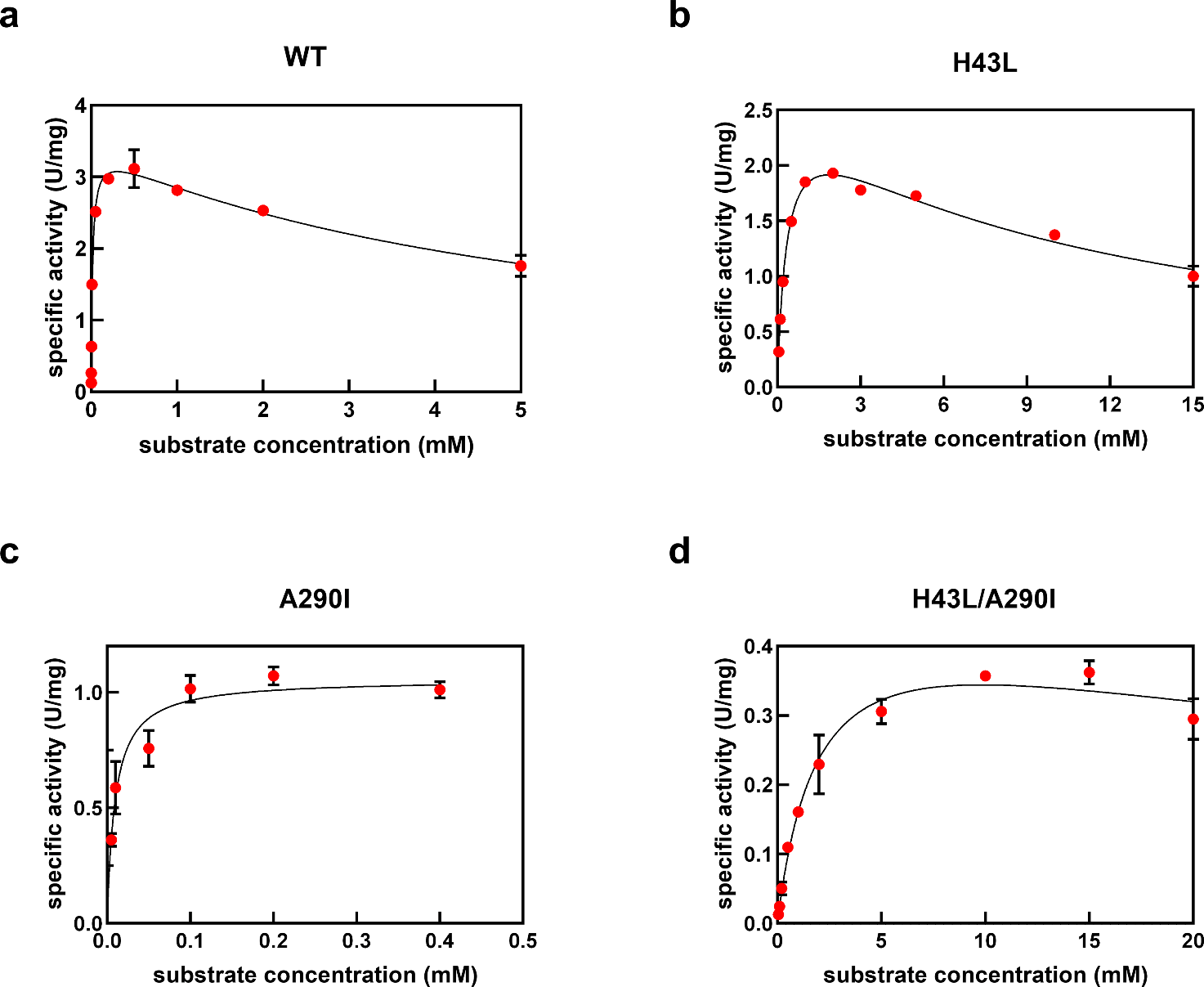


# Figure S7. Kinetic assays of *Sp*ADH2 and its mutants towards 1a while utilizing NAD^+^ according to Michaelis-Menten excess substrate inhibition equation.

Reaction conditions: c(**1a**) = 0–20 mM, c(NAD^+^) = 5 mM, 50 mM PBS (pH 7.5), 30°C, triplicate measurements. The fixed NAD^+^ concentration was chosen based on the preliminary kinetic assay to determine the *K*_M_ value of **1a** while utilizing NAD^+^. Reaction volume = 100 μL, detection volume = 200 μL, c(florescent probe) = 0.2 mM. (A). c(WT) = 0.1 mg/L, (purified enzyme). (B). c(H43L) = 0.1 mg/L, (purified enzyme). (C). c(A290I) = 0.1 mg/L, (purified enzyme). (D). c(H43L/A290I) = 0.5 mg/L, (purified enzyme).


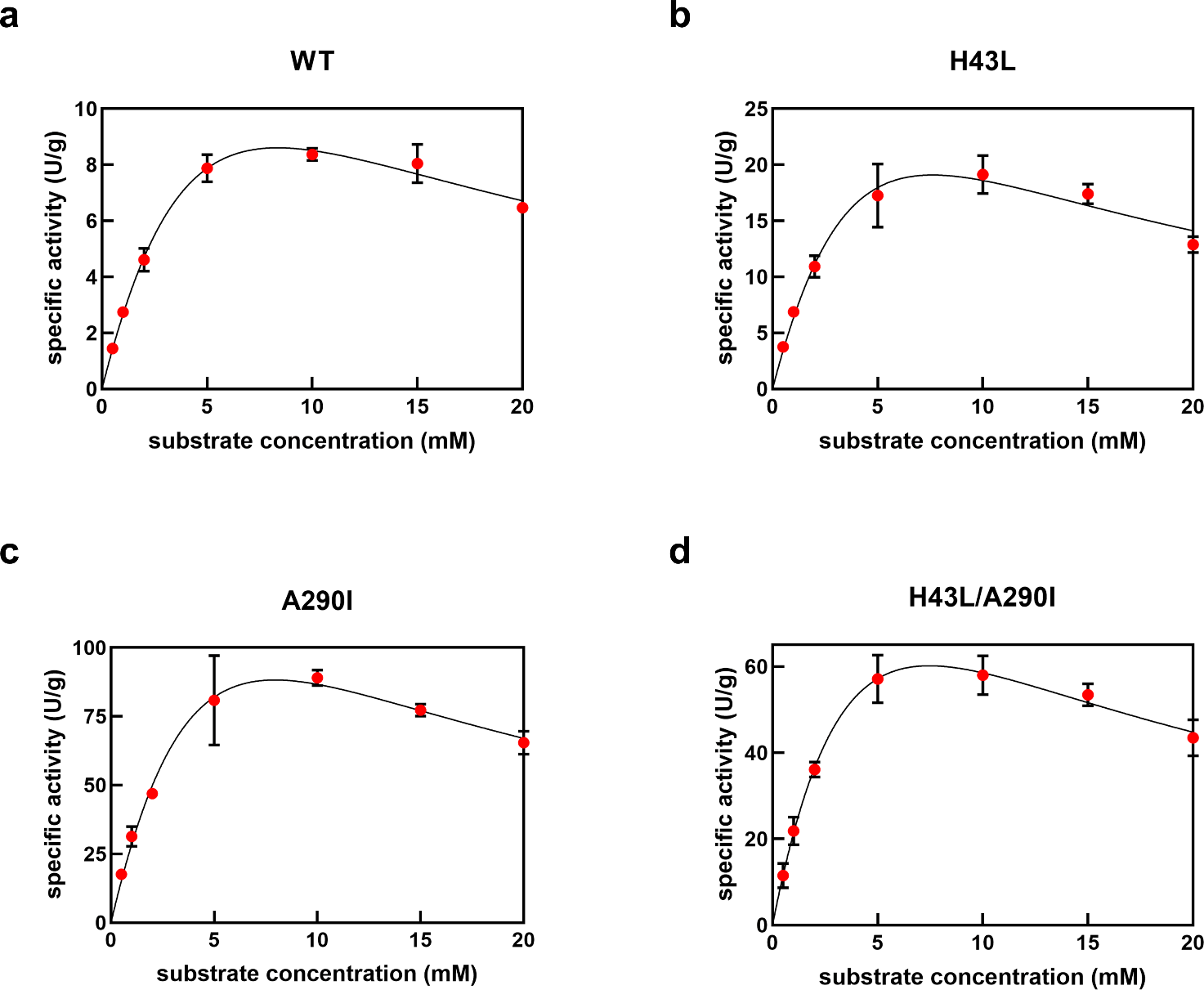


# Figure S8. Kinetic assays of *Sp*ADH2 and its mutants towards 1a while utilizing *p*-BANA^+^ according to Michaelis-Menten excess substrate inhibition equation.

Reaction conditions: c(**1a**) = 0–20 mM, c(*p*-BANA^+^) = 7.5 mM, 50 mM PBS (pH 7.5), 30°C, triplicate measurements. The fixed *p*-BANA^+^ concentration was chosen based on the preliminary kinetic assay to determine the *K*_M_ value of **1a** while utilizing *p*-BANA^+^. Reaction volume = 100 μL, detection volume = 200 μL, c(florescent probe) = 0.2 mM. (A). c(WT) = 50 mg/L, (purified enzyme). (B). c(H43L) = 50 mg/L, (purified enzyme). (C). c(A290I) = 10 mg/L, (purified enzyme). (D). c(H43L/A290I) = 10 mg/L, (purified enzyme).


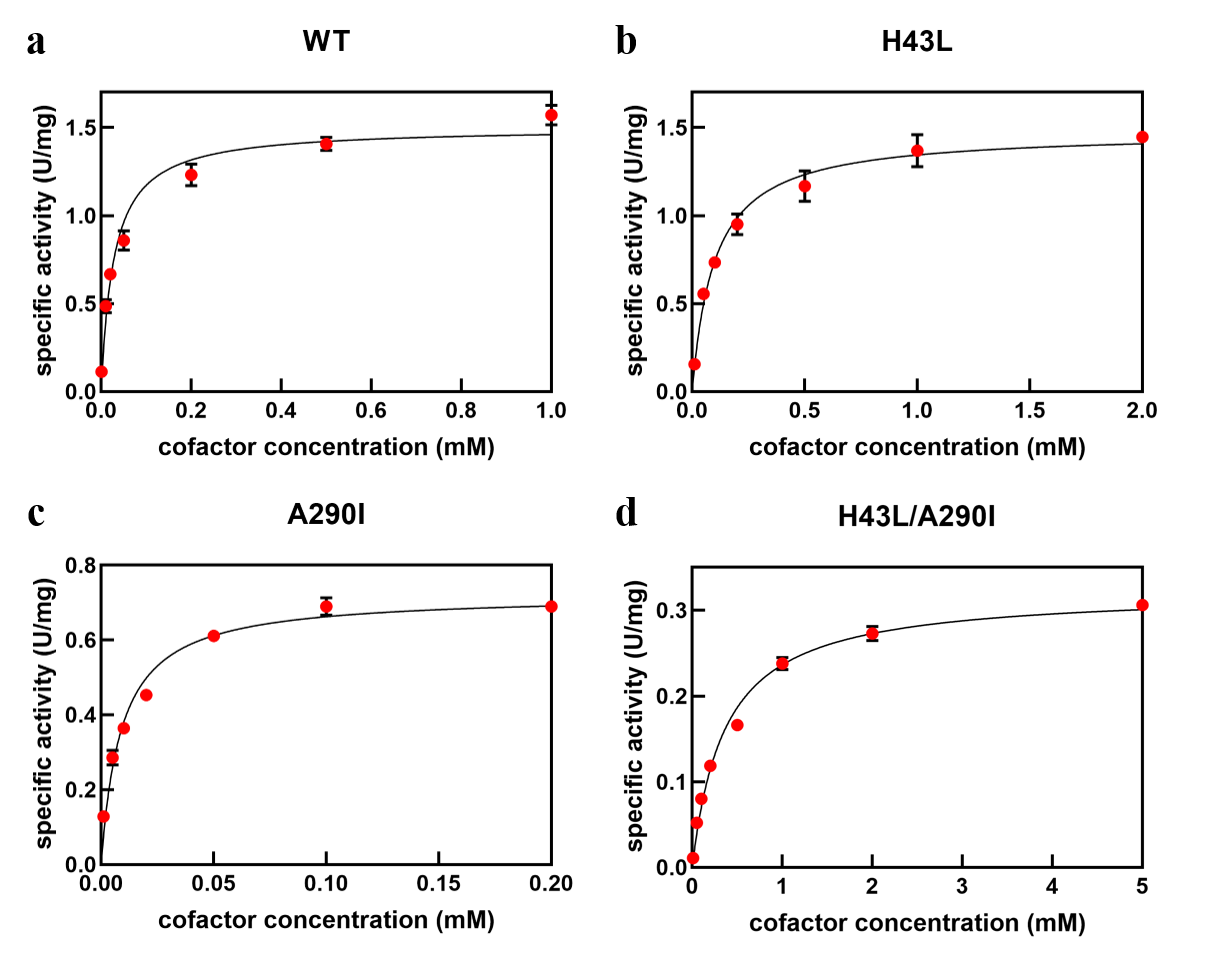


# Figure S9. Kinetic assays of *Sp*ADH2 and its mutants towards NAD^+^ while oxidizing 1a according to Michaelis-Menten equation.

Reaction conditions: c(NAD^+^) = 0–5 mM, c(**1a**) = 5 mM, 50 mM PBS (pH 7.5), 30°C, triplicate measurements. The fixed **1a** concentration was chosen based on the preliminary kinetic assay to determine the *K*_M_ value of **1a** while utilizing NAD^+^. Reaction volume = 100 μL, detection volume = 200 μL, c(florescent probe) = 0.2 mM. (A). c(WT) = 0.1 mg/L, (purified enzyme). (B). c(H43L) = 0.1 mg/L, (purified enzyme). (C). c(A290I) = 0.1 mg/L, (purified enzyme). (D). c(H43L/A290I) = 0.5 mg/L, (purified enzyme).


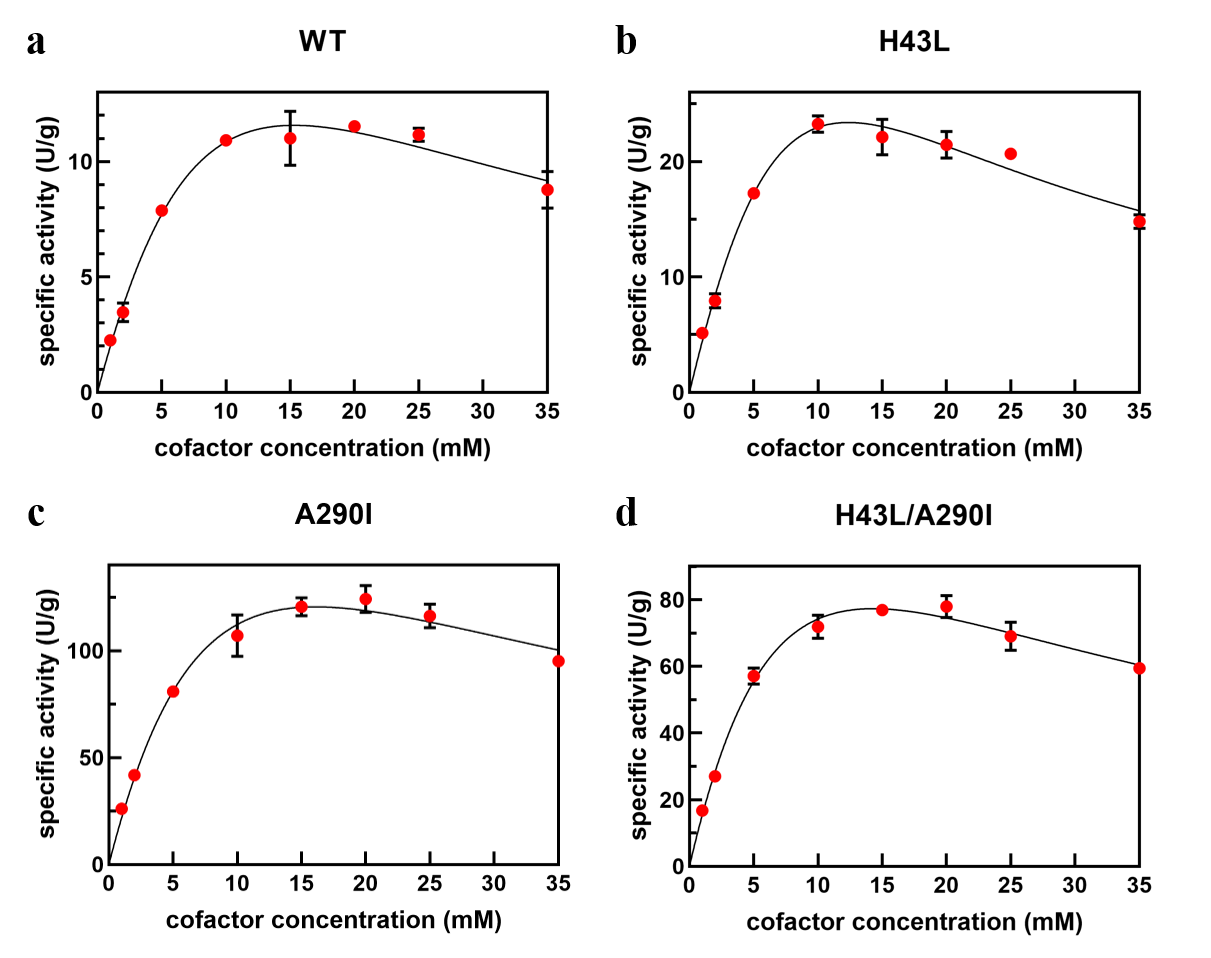


# Figure S10. Kinetic assays of *Sp*ADH2 and its mutants towards *p*-BANA^+^ while oxidizing 1a according to Michaelis-Menten excess substrate inhibition equation.

Reaction conditions: c(*p*-BANA^+^) = 0–35 mM, c(**1a**) = 5 mM, 50 mM PBS (pH 7.5), 30°C, triplicate measurements. The fixed **1a** concentration was chosen based on the preliminary kinetic assay to determine the *K*_M_ value of **1a** while utilizing *p*-BANA^+^. Reaction volume = 100 μL, detection volume = 200 μL, c(florescent probe) = 0.2 mM. (A). c(WT) = 50 mg/L, (purified enzyme). (B). c(H43L) = 50 mg/L, (purified enzyme). (C). c(A290I) = 10 mg/L, (purified enzyme). (D). c(H43L/A290I) = 10 mg/L, (purified enzyme).

# NMR

*p*-BNNA^+^

^1^H NMR (400 MHz, DMSO-*d*_6_) δ 9.75 (d, *J* = 1.6 Hz, 1H), 9.41 (dt, *J* = 6.1, 1.3 Hz, 1H), 9.05 (dt, *J* = 8.1, 1.4 Hz, 1H), 8.66 (s, 1H), 8.35 (dd, *J* = 8.1, 6.1 Hz, 1H), 8.33 – 8.25 (m, 2H), 8.21 (s, 1H), 7.90 – 7.82 (m, 2H), 6.15 (s, 2H).

^13^C NMR (101 MHz, DMSO-d_6_) δ 163.14, 148.42, 147.20, 145.80, 144.63, 141.38, 134.67, 130.83, 128.89, 124.57, 62.77.


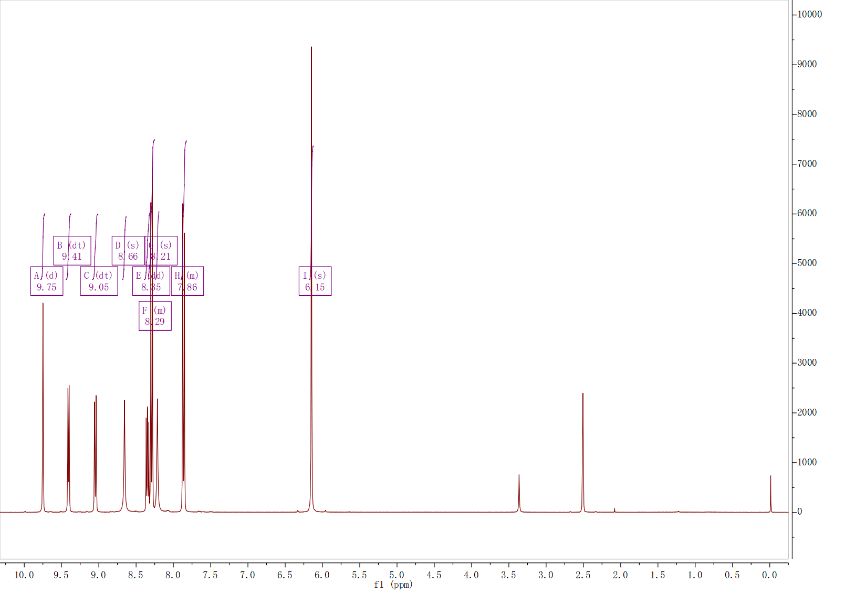


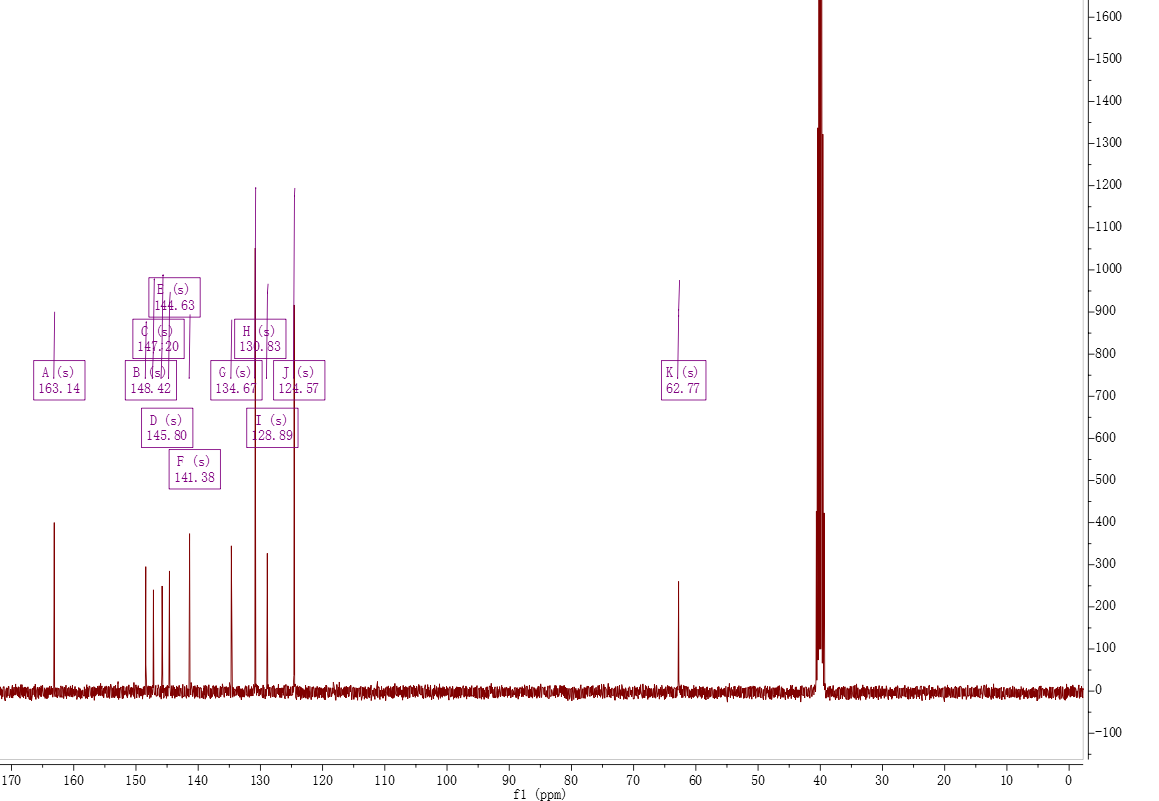


*m*-BNNA^+^

^1^H NMR (400 MHz, DMSO-*d*_6_) δ 9.78 (d, *J* = 4.2 Hz, 1H), 9.48 – 9.40 (m, 1H), 9.02 (dt, *J* = 8.1, 1.7 Hz, 1H), 8.67 – 8.58 (m, 2H), 8.37 – 8.25 (m, 2H), 8.21 (s, 1H), 8.13 (dd, *J* = 7.7, 2.9 Hz, 1H), 7.77 (t, *J* = 8.0 Hz, 1H), 6.12 (d, *J* = 4.5 Hz, 2H).

^13^C NMR (101 MHz, DMSO-d_6_) δ 163.20, 148.48, 147.00, 145.69, 144.50, 136.49, 136.06, 134.62, 131.23, 128.82, 125.03, 124.79, 62.69.


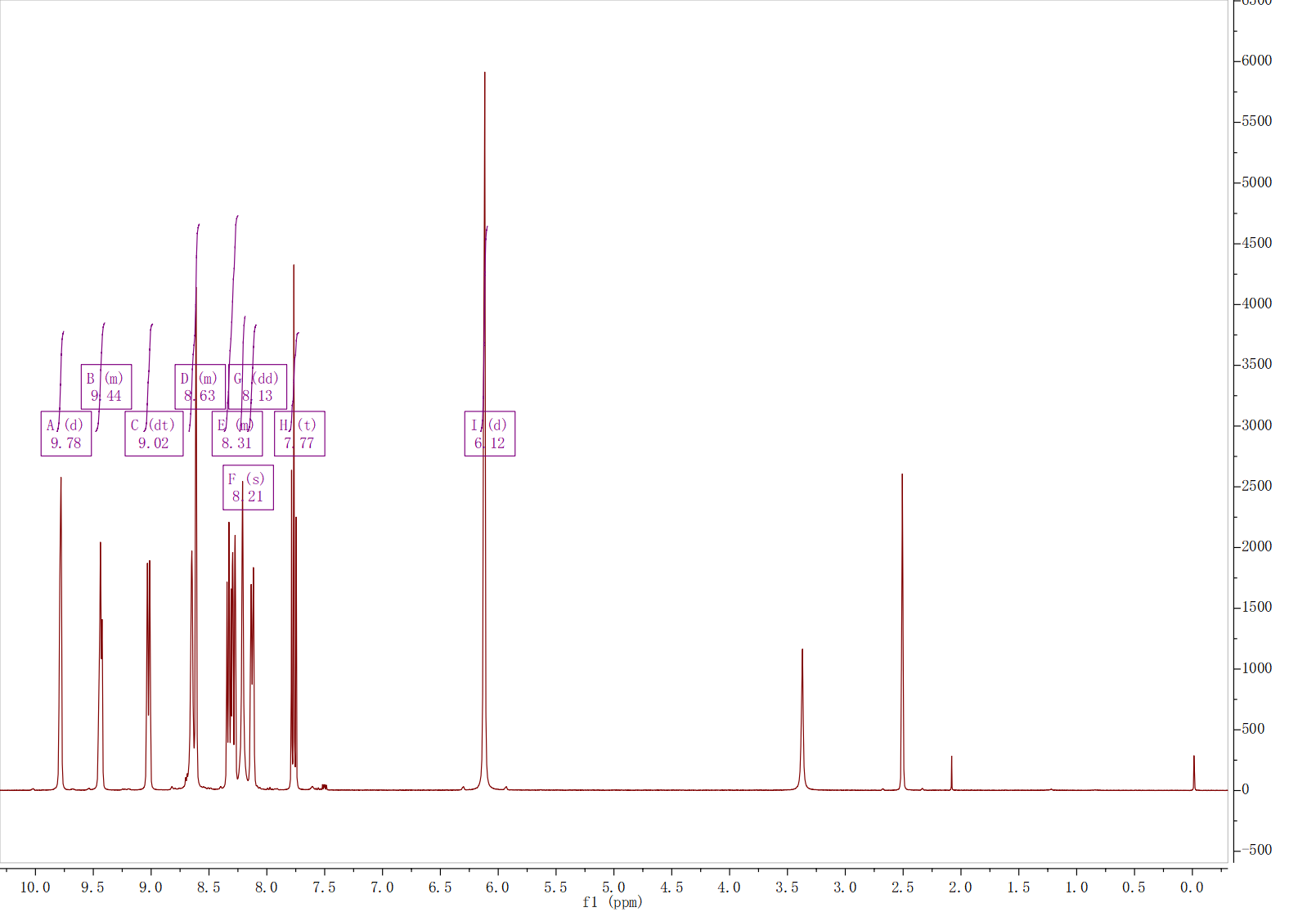


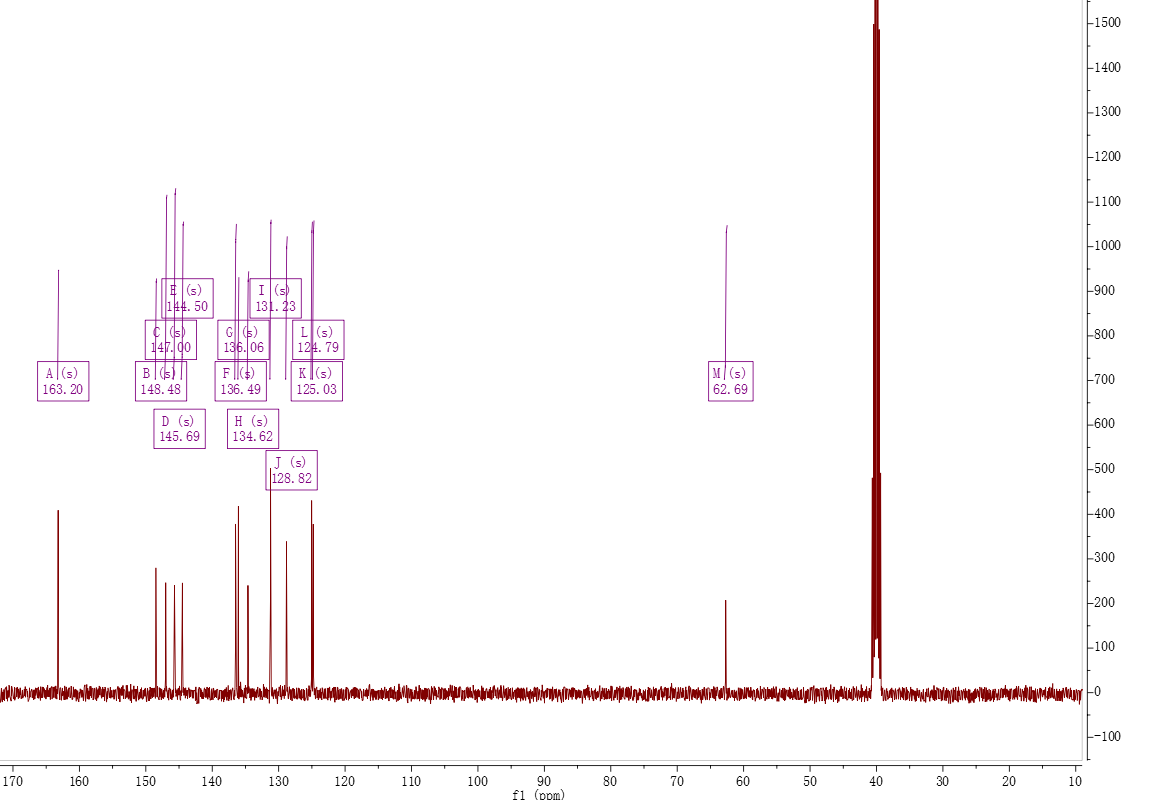


*o*-BNNA^+^

^1^H NMR (400 MHz, DMSO-*d*_6_) δ 9.59 (s, 1H), 9.26 (d, *J* = 6.1 Hz, 1H), 9.11 (dt, *J* = 8.2, 1.5 Hz, 1H), 8.68 (s, 1H), 8.38 (dd, *J* = 8.1, 6.1 Hz, 1H), 8.27 (dd, *J* = 8.1, 1.4 Hz, 1H), 8.21 (s, 1H), 7.82 (td, *J* = 7.6, 1.5 Hz, 1H), 7.75 (td, *J* = 7.7, 1.5 Hz, 1H), 7.26 (dd, *J* = 7.7, 1.5 Hz, 1H), 6.33 (s, 2H).

^13^C NMR (101 MHz, DMSO-d_6_) δ 163.17, 148.08, 147.57, 146.18, 144.68, 135.39, 134.46, 131.11, 131.06, 129.44, 128.70, 126.06, 61.37.


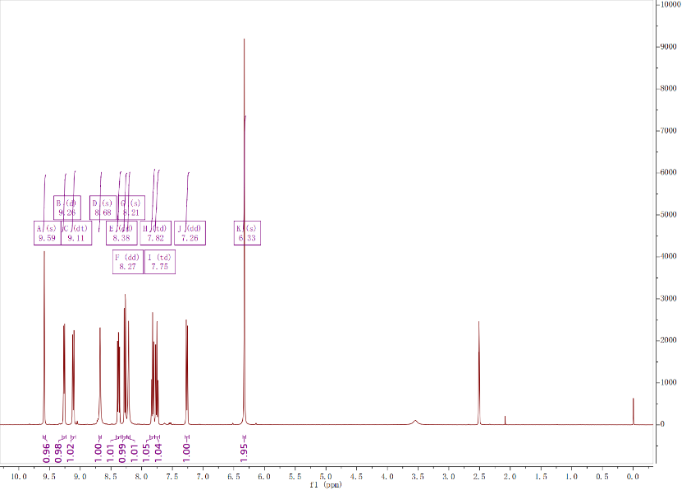


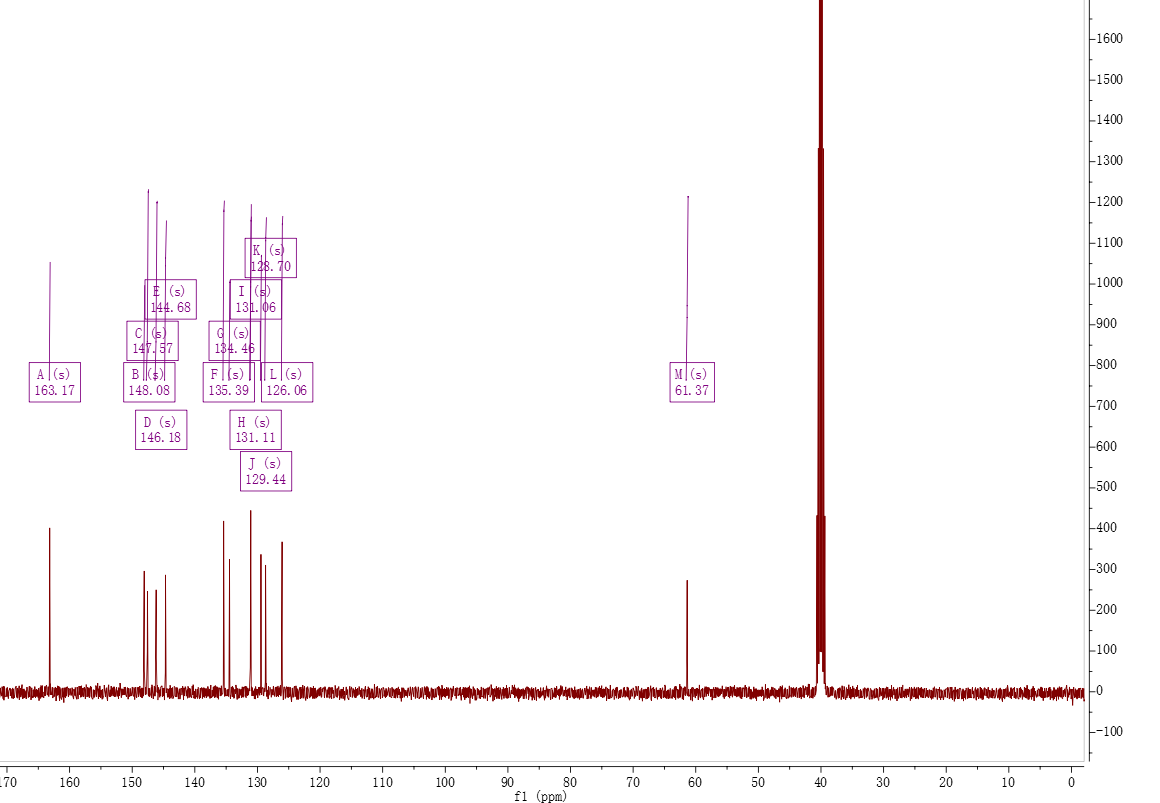


*p*-BMNA^+^

^1^H NMR (400 MHz, DMSO-*d*_6_) δ 9.69 (dd, *J* = 4.6, 2.6 Hz, 1H), 9.39 – 9.31 (m, 1H), 9.00 (dt, *J* = 8.3, 1.5 Hz, 1H), 8.66 (s, 1H), 8.29 (dd, *J* = 8.1, 6.1 Hz, 1H), 8.20 (s, 1H), 7.52 (dd, *J* = 8.1, 2.0 Hz, 2H), 7.26 (d, *J* = 7.7 Hz, 2H), 5.92 (d, *J* = 3.2 Hz, 2H), 2.30 (s, 3H).

^13^C NMR (101 MHz, DMSO-d_6_) δ 163.17, 146.74, 145.13, 144.26, 139.56, 134.47, 134.45, 131.49, 130.22, 129.61, 128.71, 63.77, 21.26, 21.23.


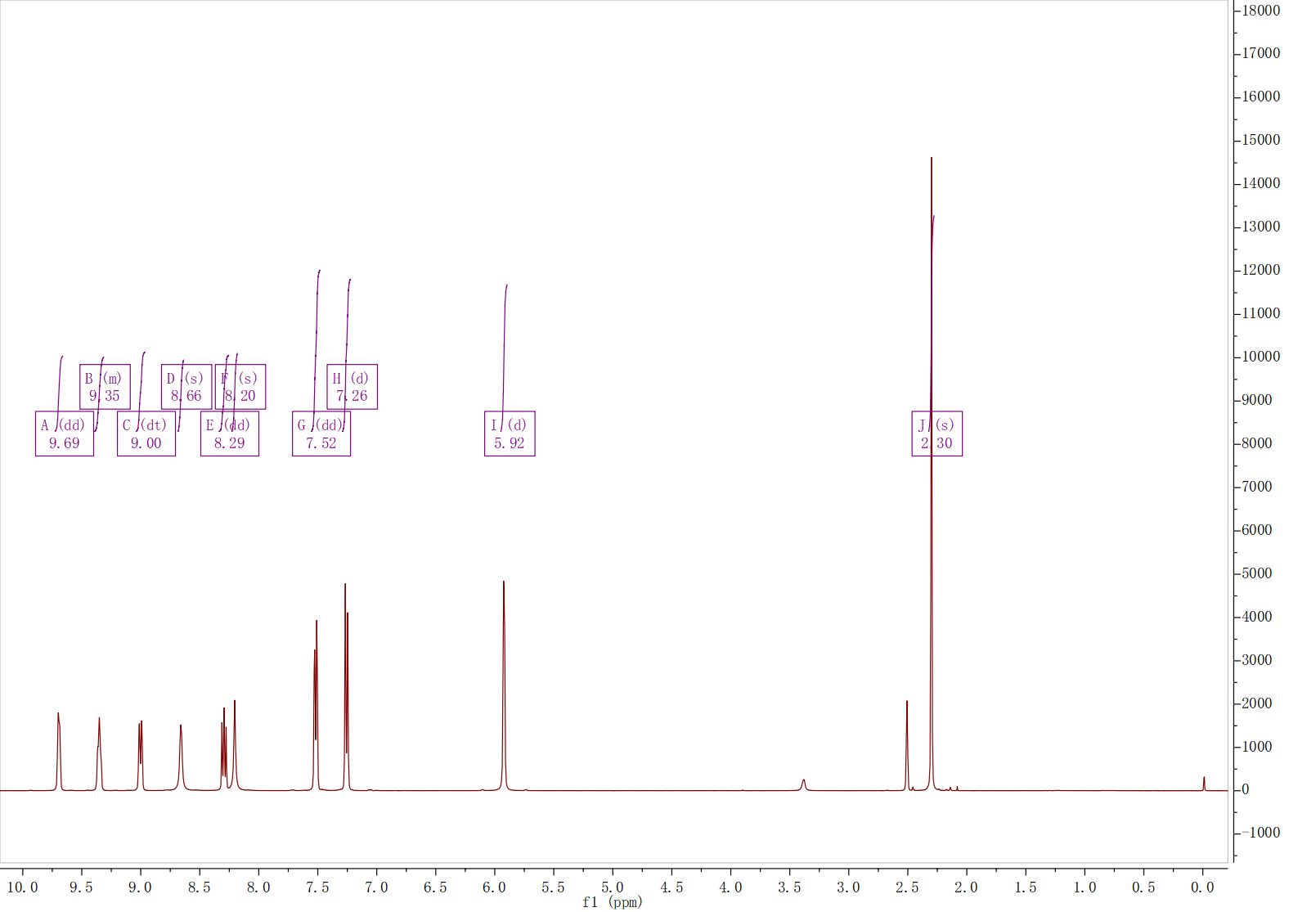


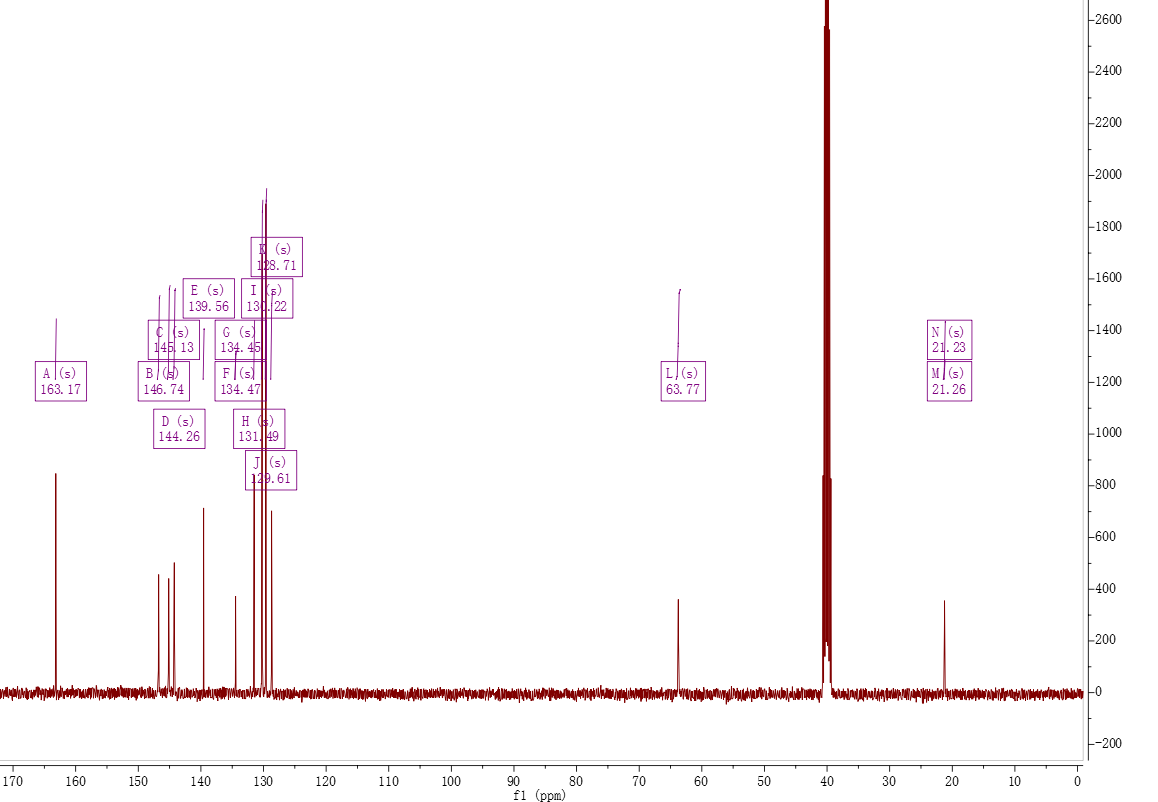


*o*-BMNA^+^

^1^H NMR (400 MHz, DMSO-*d*_6_) δ 9.54 (s, 1H), 9.18 (d, *J* = 6.1 Hz, 1H), 9.07 (d, *J* = 8.2 Hz, 1H), 8.70 (s, 1H), 8.32 (dd, *J* = 8.1, 6.1 Hz, 1H), 8.23 (s, 1H), 7.39 – 7.20 (m, 5H), 6.05 (s, 2H), 2.31 (s, 3H).

^13^C NMR (101 MHz, DMSO-d_6_) δ 163.16, 146.99, 145.44, 144.41, 137.63, 134.33, 132.46, 131.40, 129.97, 129.83, 128.73, 127.19, 62.21, 19.35.


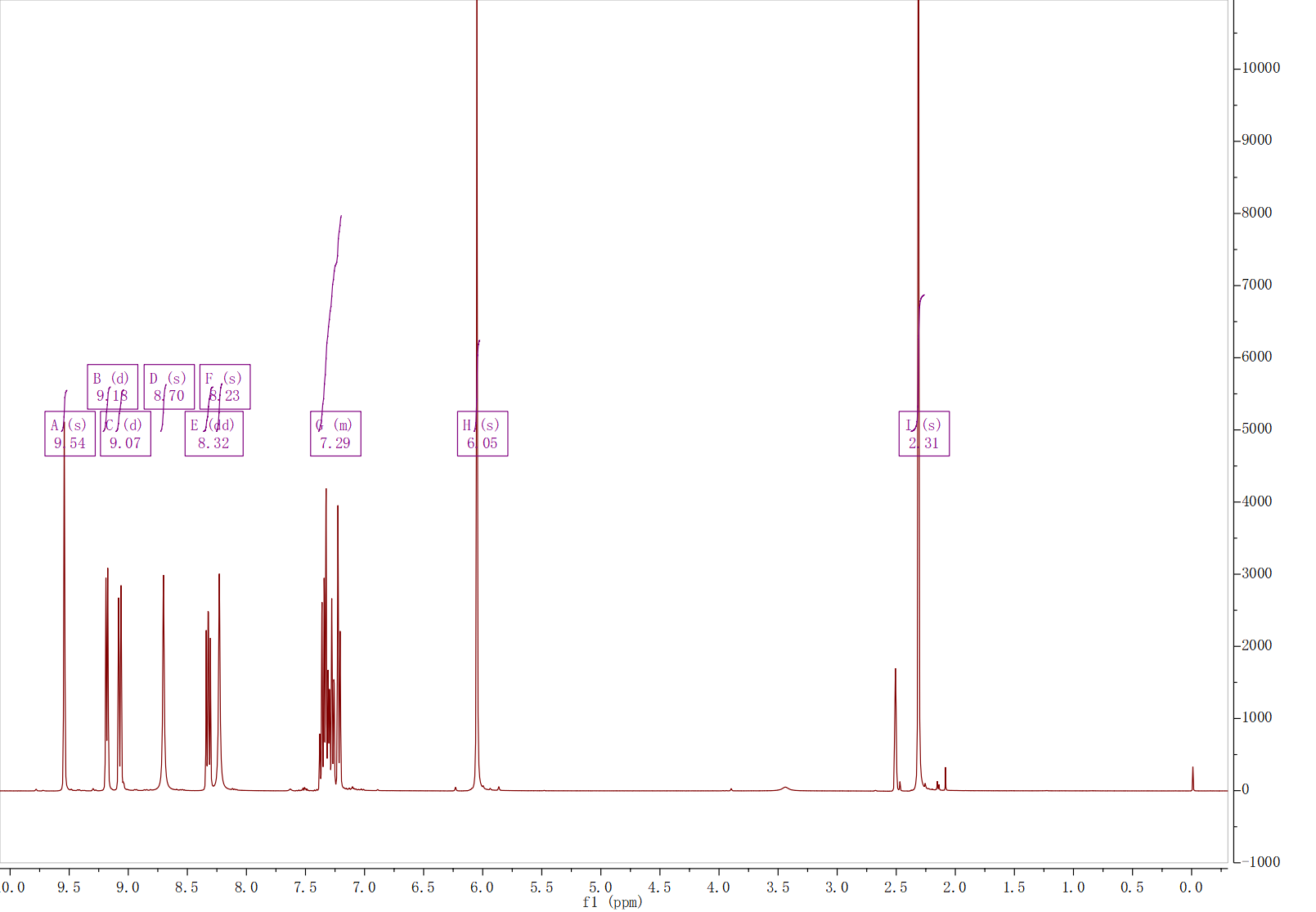


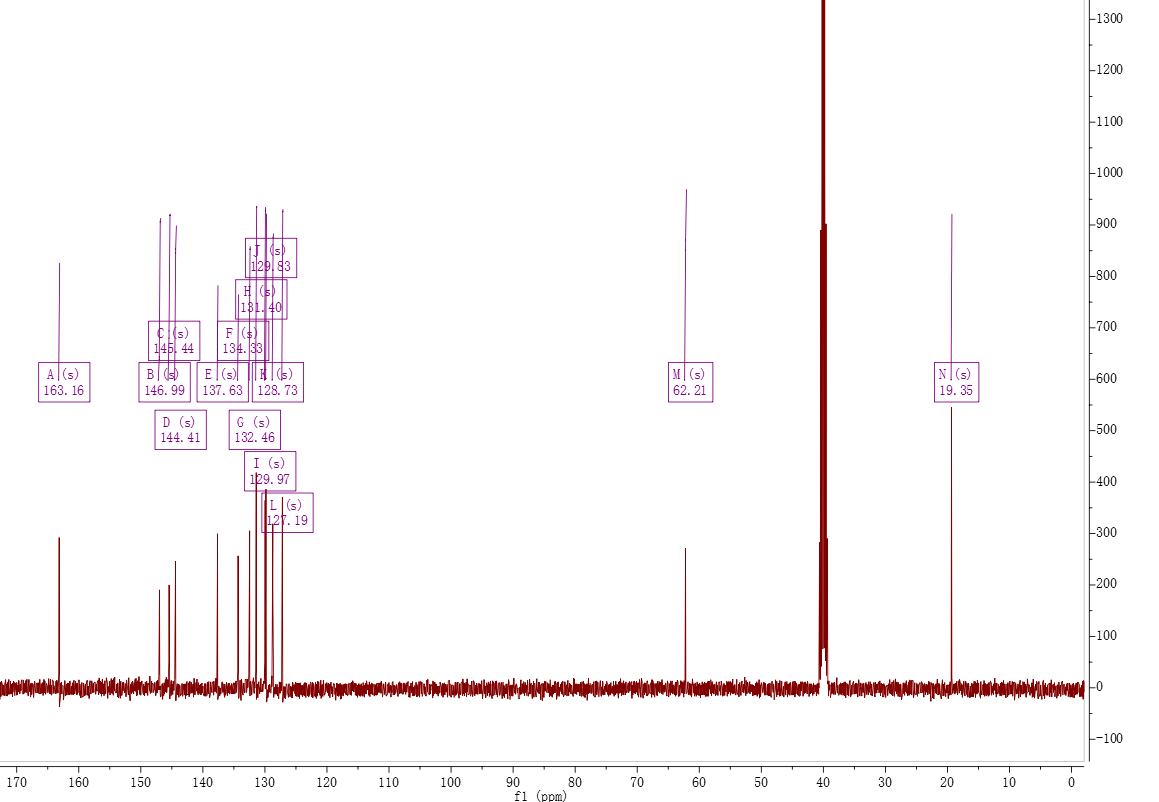


*m*-BANA^+^

^1^H NMR (400 MHz, DMSO-*d*_6_) δ 13.22 (s, 1H), 9.77 (s, 1H), 9.40 (d, *J* = 6.1 Hz, 1H), 9.03 (d, *J* = 7.7 Hz, 1H), 8.71 (s, 1H), 8.31 (dd, *J* = 8.1, 6.1 Hz, 1H), 8.21 (s, 2H), 7.99 (dd, *J* = 8.0, 1.5 Hz, 1H), 7.92 – 7.86 (m, 1H), 7.59 (t, *J* = 7.7 Hz, 1H), 6.04 (s, 2H).

^13^C NMR (101 MHz, DMSO-*d*_6_) δ 167.22, 163.17, 146.89, 145.49, 144.44, 134.85, 134.58, 134.05, 132.14, 130.72, 130.59, 130.06, 128.80, 63.42.


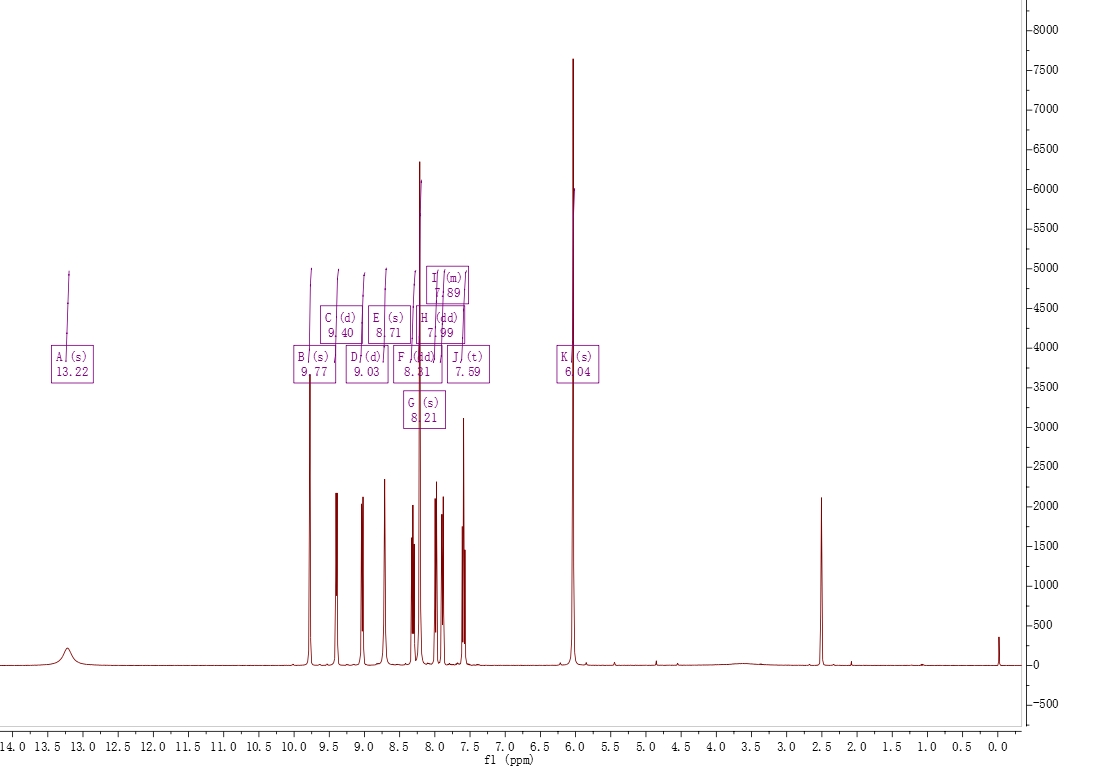


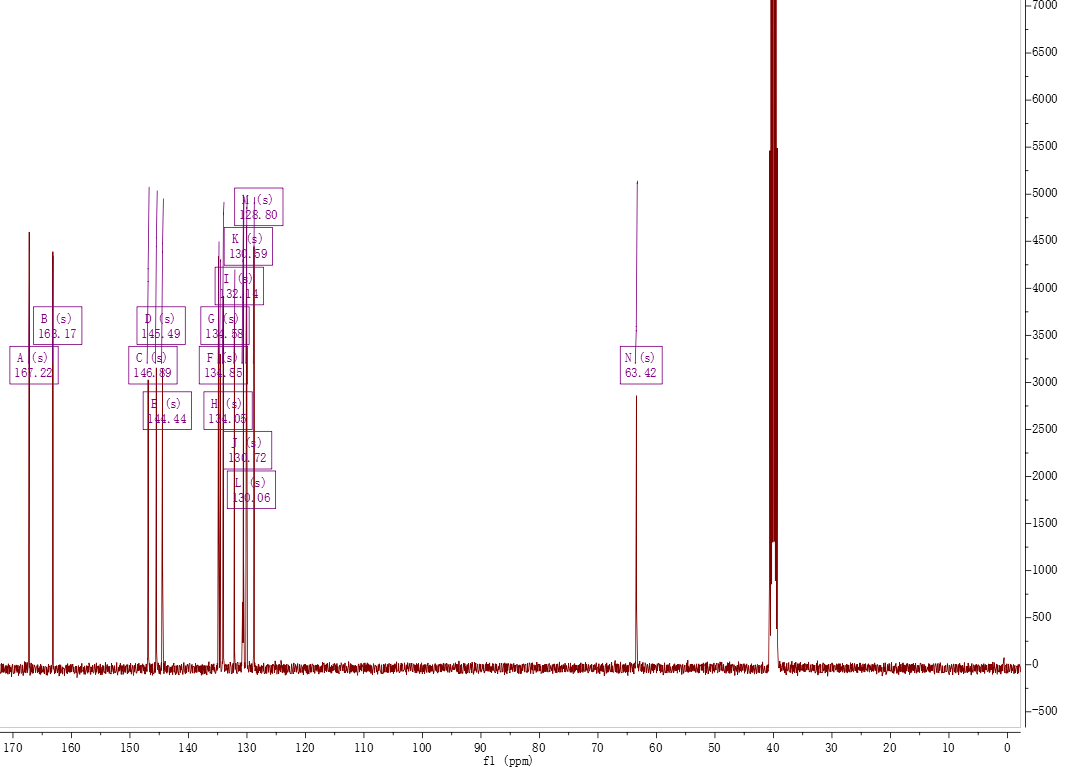


*p*-BBrNA^+^:

^1^H NMR (600 MHz, DMSO) δ 9.74 (s, 1H), 9.39 (dd, *J* = 6.2, 1.5 Hz, 1H), 9.02 (dd, *J* = 8.0, 1.8 Hz, 1H), 8.66 (s, 1H), 8.32 (dd, *J* = 8.1, 6.1 Hz, 1H), 8.23 (s, 1H), 7.94 (d, *J* = 2.2 Hz, 1H), 7.68 – 7.63 (m, 2H), 7.43 (t, *J* = 7.9 Hz, 1H), 5.97 (s, 2H).

^13^C NMR (151 MHz, DMSO) δ 162.09, 145.80, 144.33, 143.29, 143.26, 133.46, 133.44, 132.67, 131.51, 130.80, 130.78, 127.70, 122.39, 62.01, 61.97, 39.42.
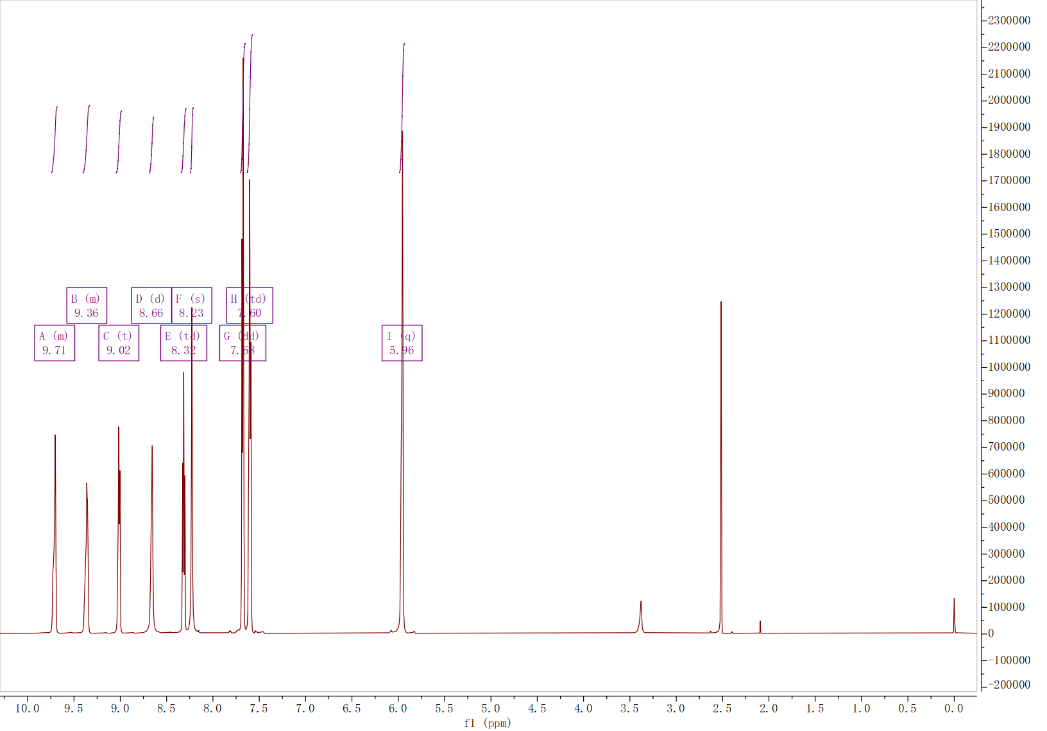

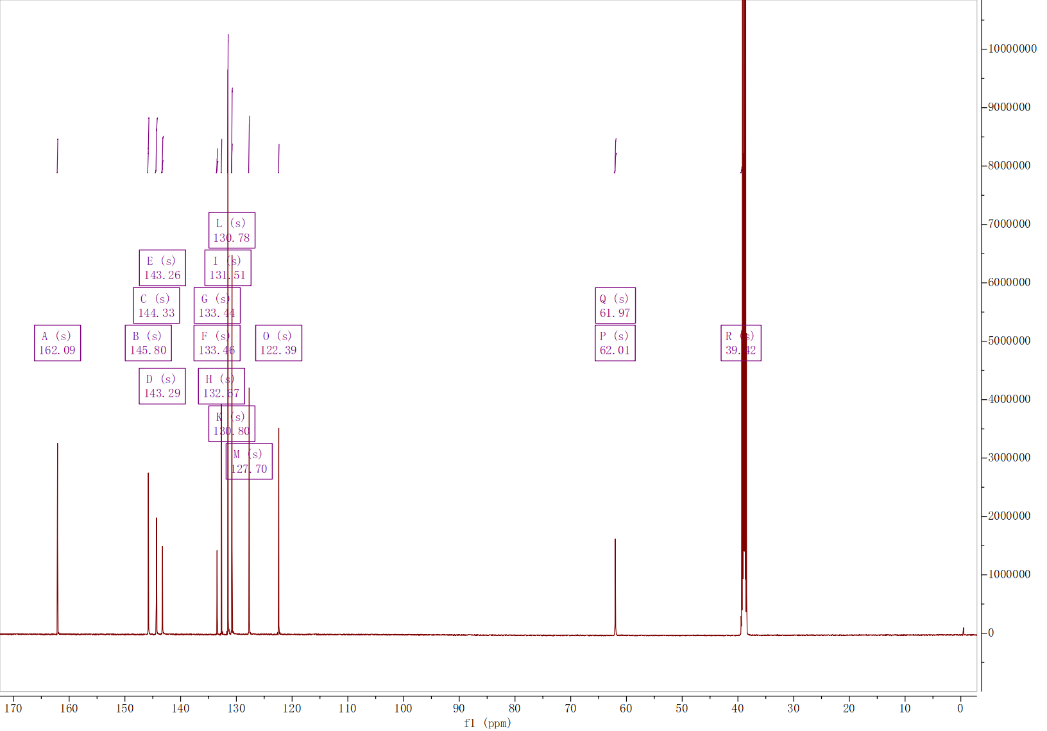


*m*-BBrNA^+^:

^1^H NMR (600 MHz, DMSO) δ 9.74 (s, 1H), 9.39 (dd, *J* = 6.2, 1.5 Hz, 1H), 9.02 (dd, *J* = 8.0, 1.8 Hz, 1H), 8.66 (s, 1H), 8.32 (dd, *J* = 8.1, 6.1 Hz, 1H), 8.23 (s, 1H), 7.94 (d, *J* = 2.2 Hz, 1H), 7.68 – 7.63 (m, 2H), 7.43 (t, *J* = 7.9 Hz, 1H), 5.97 (s, 2H).

^13^C NMR (151 MHz, DMSO) δ 163.20, 146.91, 145.51, 144.42, 136.83, 134.58, 132.83, 132.42, 131.80, 128.81, 128.76, 122.68, 62.99, 40.53.


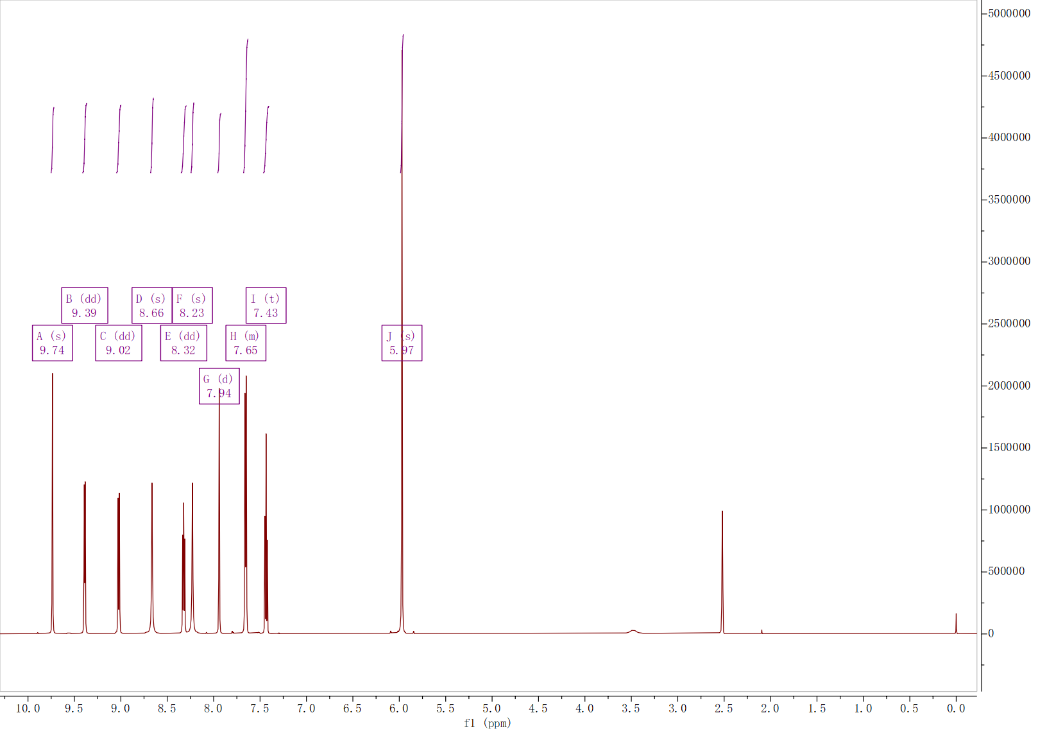

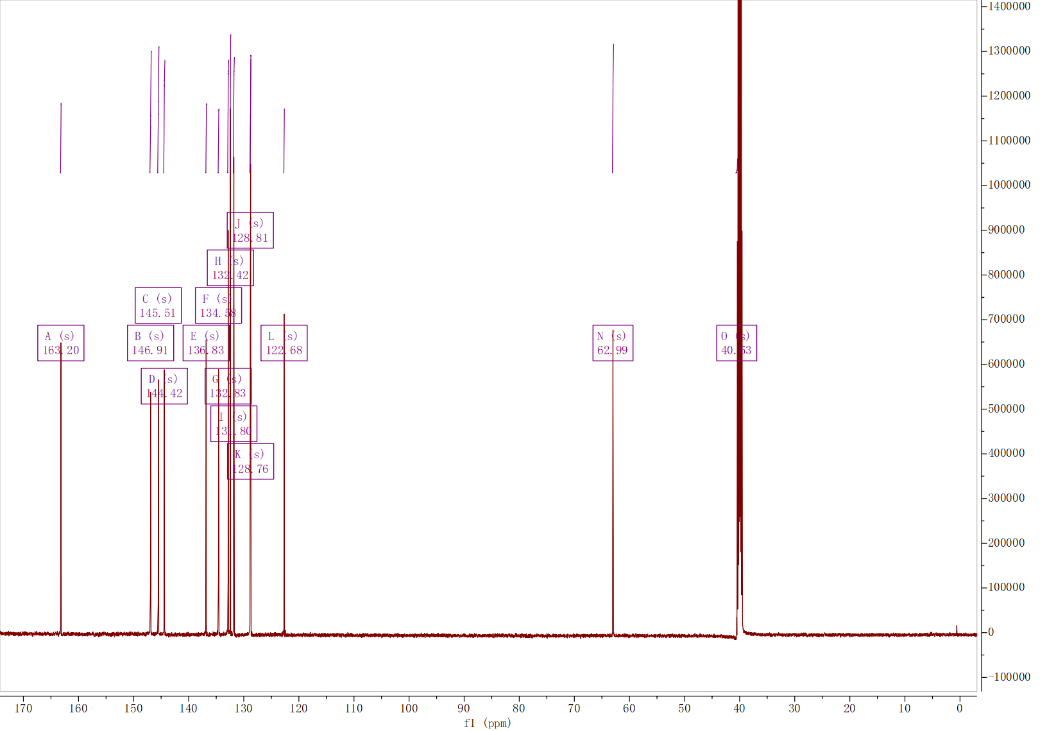


*o*-BBrNA^+^:

1H NMR (400 MHz, DMSO) δ 9.61 – 9.53 (m, 1H), 9.29 – 9.20 (m, 1H), 9.11 – 9.03 (m, 1H), 8.73 – 8.65 (m, 1H), 8.33 (ddd, J = 8.2, 6.3, 2.3 Hz, 1H), 8.24 (s, 1H), 7.61 (dt, J = 7.7, 1.9 Hz, 1H), 7.57 – 7.43 (m, 3H), 6.14 – 6.08 (m, 2H).

^13^C NMR (101 MHz, DMSO) δ 163.07, 147.28, 145.72, 144.70, 134.40, 133.75, 132.06, 132.00, 131.72, 130.56, 128.72, 128.65, 61.94.


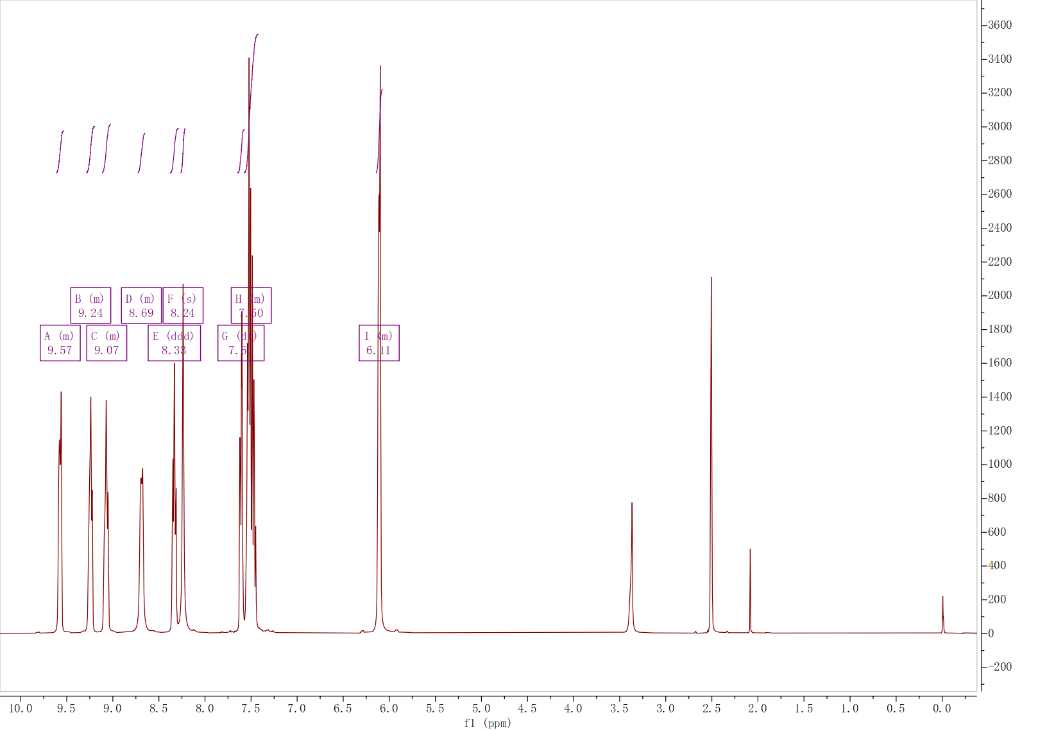

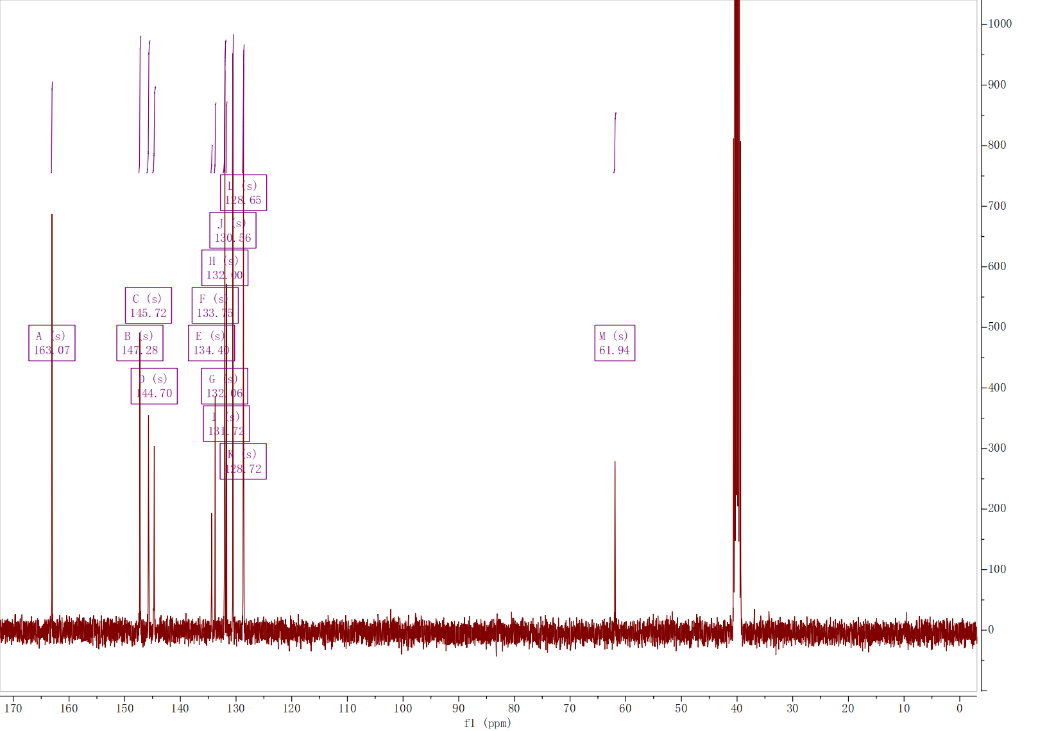


# References

Abraham MJ, Murtola T, Schulz R, Páll S, Lindahl E (2015) GROMACS: High performance molecular simulations through multi-level parallelism from laptops to supercomputers. Softwarex 1-2:19-25. <https://doi.org/10.1016/j.softx.2015.06.001>

Anandakrishnan R, Aguilar B, Onufriev AV (2012) *H*++3.0: automating p*K* prediction and the preparation of biomolecular structures for atomistic molecular modeling and simulations. Nucleic Acids Research 40:W537-W541. <https://doi.org/10.1093/nar/gks375>

Case DA, Aktulga HM, Belfon K, Cerutti DS, Cisneros GA, Cruzeiro VWD, Forouzesh N, Giese TJ, Götz AW, Gohlke H, Izadi S, Kasavajhala K, Kaymak MC, King E, Kurtzman T, Lee TS, Li PF, Liu J, Luchko T, Luo R, Manathunga M, Machado MR, Nguyen HM, O'Hearn KA, Onufriev AV, Pan F, Pantano S, Qi RX, Rahnamoun A, Risheh A, Schott-Verdugo S, Shajan A, Swails J, Wang JM, Wei HX, Wu XW, Wu YX, Zhang S, Zhao SJ, Zhu Q, Cheatham TE, III, Roe DR, Roitberg A, Simmerling C, York DM, Nagan MC, Merz KM, Jr. (2023) The AmberTools. Journal of Chemical Information and Modeling 63:6183-6191. <https://doi.org/10.1021/acs.jcim.3c01153>

Jakalian A, Jack DB, Bayly CI (2002) Fast, efficient generation of high-quality atomic charges. AM1-BCC model: II. Parameterization and validation. Journal of Computational Chemistry 23:1623-1641. <https://doi.org/10.1002/jcc.10128>

Jorgensen WL, Chandrasekhar J, Madura JD, Impey RW, Klein ML (1983) Comparison of simple potential functions for simulating liquid water. The Journal of Chemical Physics 79:926-935. <https://doi.org/10.1063/1.445869>

Li P, Merz KMJ (2016) MCPB.py: a python based metal center parameter builder. Journal of Chemical Information and Modeling 56:599-604. <https://doi.org/10.1021/acs.jcim.5b00674>

Tian C, Kasavajhala K, Belfon KAA, Raguette L, Huang H, Migues AN, Bickel J, Wang Y, Pincay J, Wu Q, Simmerling C (2020) ff19SB: Amino-acid-specific protein backbone parameters trained against quantum mechanics energy surfaces in solution. Journal of Chemical Theory and Computation 16:528-552. <https://doi.org/10.1021/acs.jctc.9b00591>

Wang J, Wolf R, Caldwell J, Kollman P, Case D (2004) Development and testing of a general amber force field. Journal of Computational Chemistry 25:1157-1174. <https://doi.org/10.1002/jcc.20035>
