# Supplementary figures and images for: Engineering a newly identified alcohol dehydrogenase from Sphingobium Sp. for efficient utilization of nicotinamide cofactors biomimetics
Source: Bioresour Bioprocess. 2025 May 5;12(1):41. doi: 10.1186/s40643-025-00870-z (PMC12052742; doi:10.1186/s40643-025-00870-z)

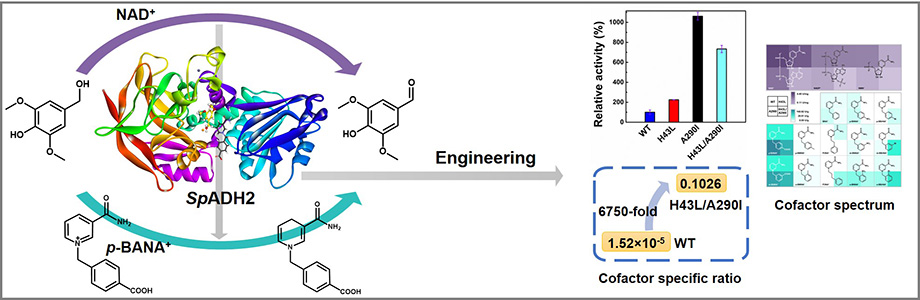

Supplement: Supplementary file 3 — Supplementary Material 3 [file 40643_2025_870_MOESM3_ESM.jpg]
